# The Timing of the Excitatory-to-Inhibitory GABA Switch Is Regulated by the Oxytocin Receptor via KCC2

**DOI:** 10.1016/j.celrep.2016.03.013

**Published:** 2016-03-24

**Authors:** Marianna Leonzino, Marta Busnelli, Flavia Antonucci, Claudia Verderio, Michele Mazzanti, Bice Chini

**Affiliations:** 1 Institute of Neuroscience, Consiglio Nazionale delle Ricerche, Milan 20129, Italy; 2 Department of Biotechnology and Translational Medicine, University of Milan, Milan 20129, Italy; 3 Humanitas Clinical and Research Center, IRCCS, Rozzano, Milan 20089, Italy; 4 Department of Bioscience, University of Milan, Milan 20133, Italy

## Abstract

Oxytocin and its receptor (Oxtr) play a crucial role in the postnatal transition of neuronal GABA neurotransmission from excitatory to inhibitory, a developmental process known as the GABA switch. Using hippocampal neurons from Oxtr-null mice, we show that (1) Oxtr is necessary for the correct timing of the GABA switch by upregulating activity of the chloride cotransporter KCC2, (2) Oxtr, in a very early and narrow time window, directly modulates the functional activity of KCC2 by promoting its phosphorylation and insertion/stabilization at the neuronal surface, and (3) in the absence of Oxtr, electrophysiological alterations are recorded in mature neurons, a finding consistent with a reduced level of KCC2 and increased susceptibility to seizures observed in adult Oxtr-null mice. These data identify KCC2 as a key target of oxytocin in postnatal events that may be linked to pathogenesis of neurodevelopmental disorders.

## Highlights

• Oxtr is necessary for the correct timing of the GABA switch in developing neurons
• Oxtr modulates the GABA switch by directly regulating the Cl^−^ transporter KCC2
• OXT actions on KCC2 are restricted to an early and narrow time window
• Oxtr deficit causes long-lasting defects in excitation/inhibition balance in neurons

## Graphical Abstract

**Figure:**
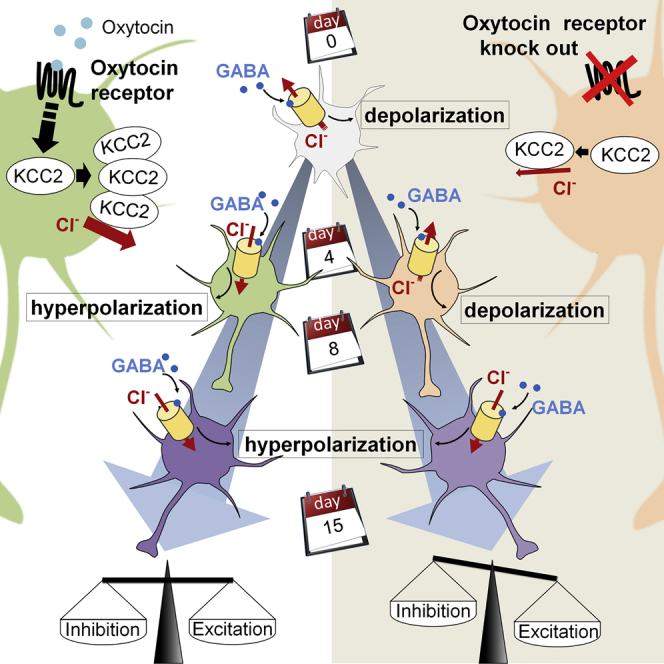

## Teaser

Dysfunctions of the oxytocin system can contribute to neurodevelopmental disorders. Leonzino et al. find that in the absence of the oxytocin receptor, the chloride transporter KCC2 is downregulated, causing a delayed excitatory-to-inhibitory GABA switch and long-lasting electrophysiological alterations in neurons.

## Introduction

To correctly shape neuronal circuits, postnatal brain development requires a finely tuned balance between excitation and inhibition (E/I). Impairments of this balance have been proposed to underlie many neurodevelopmental brain disorders including autism. The most critical determinants of this balance are glutamate and γ-aminobutyric acid (GABA), respectively the main excitatory and inhibitory neurotransmitters. At early stages of development, however, activation of GABA_A_ receptors (GABA_A_R) generates membrane depolarization and thus excitation. Therefore, in immature neurons, both glutamate and GABA, by inducing depolarization and Ca^2+^ influx through voltage-operated Ca^2+^ channels (VOCC), work in synergy on proliferation, migration, maturation, and differentiation. As a consequence, the proper timing of GABA transition from depolarizing to hyperpolarizing is fundamental for a correct development of the brain (Ben-Ari et al., 1989).

The switch in GABA polarity has been shown to have a biphasic time course: the first phase is an abrupt and fully reversed switch that is temporally restricted to the delivery period (Tyzio et al., 2006); the second, most commonly referred to as “GABA switch,” is a progressive and permanent switch that starts soon after birth and is complete, in rodents, by the end of the first postnatal week (Valeeva et al., 2013). The GABA switch relies on a developmentally regulated expression of the Na^+^-K^+^-2Cl^−^ cotransporter 1 (NKCC1) and the K^+^-Cl^−^ cotransporter 2 (KCC2). Due to a high level of NKCC1, a Cl^−^ importer, immature neurons accumulate this anion and, upon GABA_A_Rs opening, Cl^−^ efflux generates membrane depolarization. Conversely, mature neurons express higher levels of the Cl^−^ exporter KCC2 and the resulting lower intracellular Cl^−^ concentration drives Cl^−^ influx through GABA_A_Rs and leads to hyperpolarization (Rivera et al., 1999). While the molecular players of the GABA switch are well characterized, the signals that trigger this event have been only partially clarified.

Oxytocin (Oxt), a hypothalamic neurohormone known for decades for promoting parturition and lactation and for its role in social behavior (Meyer-Lindenberg et al., 2011), has also been implicated in the GABA switch. Maternal Oxt was reported to regulate the first transient phase of the GABA switch in the newborns (Tyzio et al., 2006). Pre-delivery treatments with Oxt or with a selective NKCC1 inhibitor in a model of autism, the valproate rat, and in a model of fragile X, the *Fmr1*^−/−^ mouse, were shown to rescue the altered GABA balance in pups and social behavioral deficits in adults (Tyzio et al., 2014). However, it is at present unknown if Oxt also plays a role on the postnatal phase of the GABA switch. To address this issue, we took advantage of the *Oxtr*^−/−^ mouse, a genetic model devoid of the oxytocin receptor (Oxtr), the main molecular target of Oxt in the brain. We have previously shown that *Oxtr*^−/−^ animals display an autistic-like phenotype, which includes social and cognitive deficits and increased susceptibility to seizures, compatible with an altered E/I balance (Sala et al., 2011). Our present findings indicate that Oxtr is indeed essential for the proper developmental increase of KCC2 and for the consequent switch in GABA activity. In particular, we found that Oxt directly modulates the insertion of KCC2 at the plasma membrane in an early and very narrow developmental time window, thus affecting the GABA switch and neuronal excitability in a critical period for neuronal maturation.

## Results

### Delayed GABA Switch in *Oxtr*^−/−^ Hippocampal Neurons

**Figure 1:**
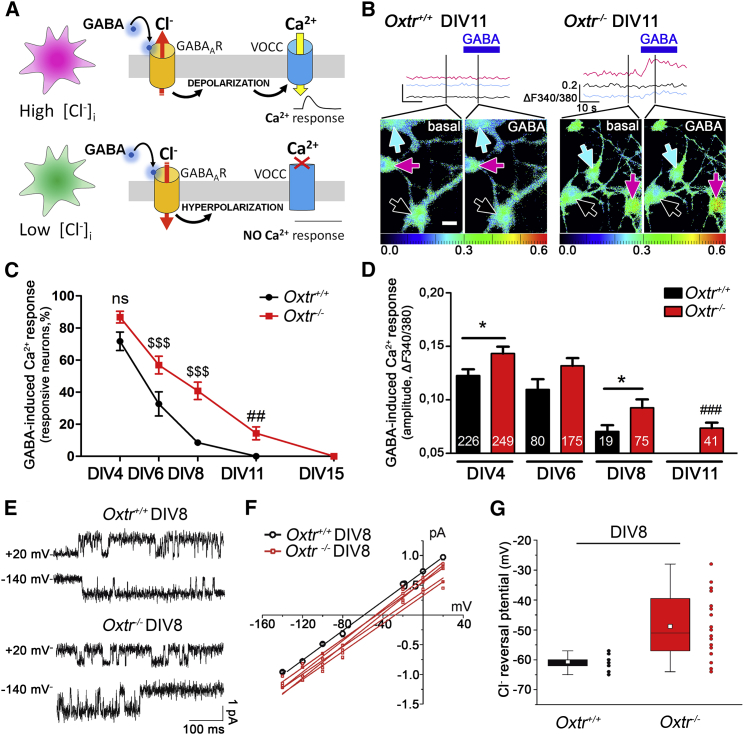
*Oxtr*^−/−^ Neurons Have a Delayed GABA Switch (A) Schematic representation of depolarizing and hyperpolarizing GABA_A_R-induced responses monitored by Ca^2+^ imaging. In neurons with high [Cl^−^]_i_ (pink), GABA-induced depolarization opens VOCCs, leading to a Ca^2+^ peak. In neurons with low [Cl^−^]_i_ (green), GABA determines hyperpolarization, VOCCs remain closed, and no Ca^2+^ response is induced. (B) Representative traces of [Ca^2+^]_i_ variations in the soma of DIV11 neurons in basal conditions and upon 100 μM GABA administration. Each trace refers to the recorded cell pointed by the color-matched arrow; pseudocolor scale from blue to red indicates increasing [Ca^2+^]. Scale bar represents 10 μm. (C) Percentage of *Oxtr*^*+/+*^ and *Oxtr*^−/−^ neurons showing GABA-induced Ca^2+^ responses along development. Data are from at least two different preparations (five to ten coverslips). (D) Amplitude of GABA-induced Ca^2+^ peaks in *Oxtr*^*+/+*^ and *Oxtr*^*−/−*^ neurons at different stages of development. Number of responsive cells is reported in the bars. See Figure S1A for KCl-induced responses. (E–G) Cell-attached recordings of single GABA_A_ receptor in *Oxtr*^*+/+*^ and *Oxtr*^−/−^ hippocampal neuron at DIV8. (E) Single channel current traces at +20 and −140 mV membrane potential in *Oxtr*^*+/+*^ (top) and *Oxtr*^−/−^ (bottom) neurons. (F) i/V relationship of average *Oxtr*^*+/+*^ single-channel recordings (n = 8; conductance 12 ± 0.2 pS) and five single experiments from *Oxtr*^−/−^ neurons (average conductance 12 ± 0.6). Current reversal potential, obtained by linear fitting of the experimental data, was adjusted in each cell according to the measured resting membrane potential. (G) Chart plot of single-channel current reversal potential for *Oxtr*^*+/+*^ (n = 14) and *Oxtr*^−/−^ (n = 22) neurons. See Figure S1B for resting membrane potentials at DIV8. Data are presented as mean ± SEM; two-way ANOVA (Bonferroni post hoc test, ^$$^p < 0.01 and ^$$$^p < 0.001; one-sample t test, ^##^p < 0.01 and ^###^p < 0.001; Student’s t test, ^∗^p < 0.05; and Student’s t test with Welch’s correction, ^&&&^p < 0.0001). All mean, SEM, n, and p values are listed in Table S1.

The timing of the GABA switch can be monitored, in developing neuronal cultures, by measuring the occurrence and amplitude of GABA-induced Ca^2+^ responses (Figures 1A and 1B). To disclose any temporal difference in the occurrence of the GABA switch between *Oxtr*^−/−^ and *Oxtr*^*+/+*^ cultures, we evaluated, during development, the percentage of neurons showing GABA-induced Ca^2+^ transients and the amplitude of such responses (Figures 1C and 1D). At all time points, we found a significantly larger proportion of *Oxtr*^−/−^ neurons increasing Ca^2+^ upon GABA stimulation (two-way ANOVA: genotype effect, F(1,67) = 28.95, p < 0.0001; time effect, F(4,67) = 86.51, p < 0.0001). Most interestingly, in *Oxtr*^*+/+*^ neurons, the GABA-induced Ca^2+^ transients were completely lost at days in vitro 11 (DIV11), whereas in *Oxtr*^−/−^ neurons, they disappeared only at DIV15 (Figure 1C).

Moreover, the amplitude of the Ca^2+^ responses was significantly higher in *Oxtr*^−/−^ than in *Oxtr*^*+/+*^ neurons (Figure 1D; two-way ANOVA: genotype effect, F(1,827) = 5.56, p = 0.0186; time effect, F(2,827) = 11.41, p < 0.0001), suggesting a stronger Cl^−^ gradient. A greater amplitude of Ca^2+^ responses could be caused also by an increased VOCC expression. However, this possibility can be excluded in *Oxtr*^−/−^ neurons, since Ca^2+^ transients evoked by the administration of KCl (50 mM) were not augmented and were significantly reduced at DIV8 and DIV11 (Figure S1A).

Consistent with calcium measurements, cell-attached recordings of single GABA_A_ receptor at DIV8 showed a difference in the reversal potential between *Oxtr*^*+/+*^ and *Oxtr*^−/−^ (Figures 1E and 1F). The range of GABA_A_ reversal potential was significantly different in *Oxtr*^−/−^ neurons (between −30 and −60 mV) than in *Oxtr*^*+/+*^ (between −57 and −65 mV; t test with Welch’s correction, p < 0.0001) (Figure 1G), even in the presence of a not significantly different resting membrane potential in the two populations (Figure S1B).

Altogether, these data indicate that in *Oxtr*^−/−^ neurons, the GABA switch is delayed.

### Impaired KCC2 Upregulation and Phosphorylation in *Oxtr*^−/−^ Neurons

**Figure 2:**
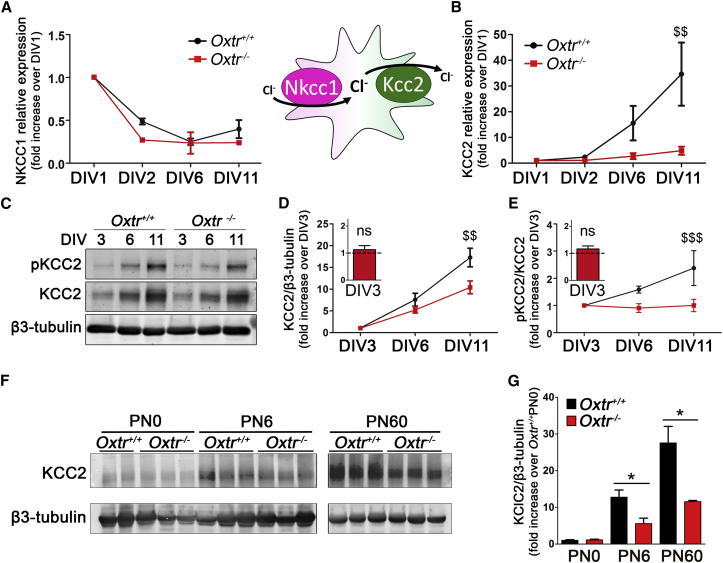
*Oxtr*^−/−^ Neurons Have Impaired KCC2 Expression (A and B) NKCC1 (A) and KCC2 (B) transcript levels during in vitro development measured by real-time qPCR analysis in three or four independent preparations and normalized on DIV1. The cartoon depicts the directions of Cl^−^ transport by NKCC1 and KCC2. (C) Representative immunoblot of neuronal lysates at DIV3, DIV6, and DIV11 probed for p-Ser940KCC2 (pKCC2) and total KCC2. (D and E) Quantification of total KCC2 normalized on β3-tubulin (D; n = 6) and of pKCC2 normalized on total KCC2 (E; n = 9) shown as fold increase over DIV3. Insets show no difference at DIV3 between *Oxtr*^−/−^ (red bars) and *Oxtr*^*+/+*^ (dotted lines). (F and G) Immunoblot (F) and relative quantification (G) of hippocampal KCC2 expression at PN0, PN6, and PN60. Data are presented as mean ± SEM; two-way ANOVA (Bonferroni post hoc test, ^$$^p < 0.01 and ^$$$^p < 0.001; one-sample t test, ^##^p < 0.01 and ^###^p < 0.001; and Student’s t test, ^∗^p < 0.05). All mean, SEM, n, and p values are listed in Table S2.

In developing neurons, downregulation of NKCC1 and upregulation of KCC2 both contribute to reduce [Cl^−^]_i_; as a consequence, altered KCC2 and/or NKCC1 expression can be responsible for the delayed GABA switch in *Oxtr*^−/−^ neurons. We thus investigated by real-time qPCR the expression profile of these transporters in *Oxtr*^−/−^ and *Oxtr*^*+/+*^ neurons from DIV1 to DIV11 (Figures 2A and 2B). The NKCC1 transcript was similarly downregulated in the two neuronal cultures (Figure 2A; two-way ANOVA: genotype effect, (F(1,14) = 4.13, p > 0.05). On the contrary, the KCC2 transcript underwent strong upregulation (30-fold) in *Oxtr*^*+/+*^ neurons, while it barely increased by 5-fold in *Oxtr*^−/−^ neurons (Figure 2B; two-way ANOVA: genotype effect, (F(1,23) = 8.91, p = 0.0066). Moreover, reduced KCC2 protein expression was observed in cultured *Oxtr*^−/−^ neurons (Figures 2C and 2D; two-way ANOVA: genotype effect, F(1,29) = 9.37, p < 0.0047). These findings point to a defect in KCC2 upregulation as a main factor for the delayed GABA switch in *Oxtr*^−/−^ neurons. Consistently, a KCC2 deficit was also found in hippocampal tissues from postnatal day 6 (PN6) and PN60 *Oxtr*^−/−^ mice (Figures 2F and 2G).

We then analyzed KCC2 phosphorylation at Ser940 (pKCC2), a post-translational modification that stabilizes KCC2 at the cell surface and correlates with its cellular activity (Lee et al., 2007). We found a significant reduction of pKCC2 in *Oxtr*^−/−^ versus *Oxtr*^*+/+*^ neurons (Figures 2C and 2E; two-way ANOVA: genotype effect, F(1,45) = 13.63, p = 0.0006). The ratio between pKCC2 and KCC2 (Figure 2E) is an index of the amount of cotransporter at the plasma membrane. Between DIV3 and DIV11, this ratio underwent a 2-fold increase in *Oxtr*^*+/+*^ neurons, whereas it remained constant in *Oxtr*^−/−^ cells. These data suggest that Oxtr deficits affect both KCC2 expression and phosphorylation.

### Oxt Promoted Phosphorylation, Plasma Membrane Expression, and Function of KCC2 in a Restricted Developmental Time Window

**Figure 3:**
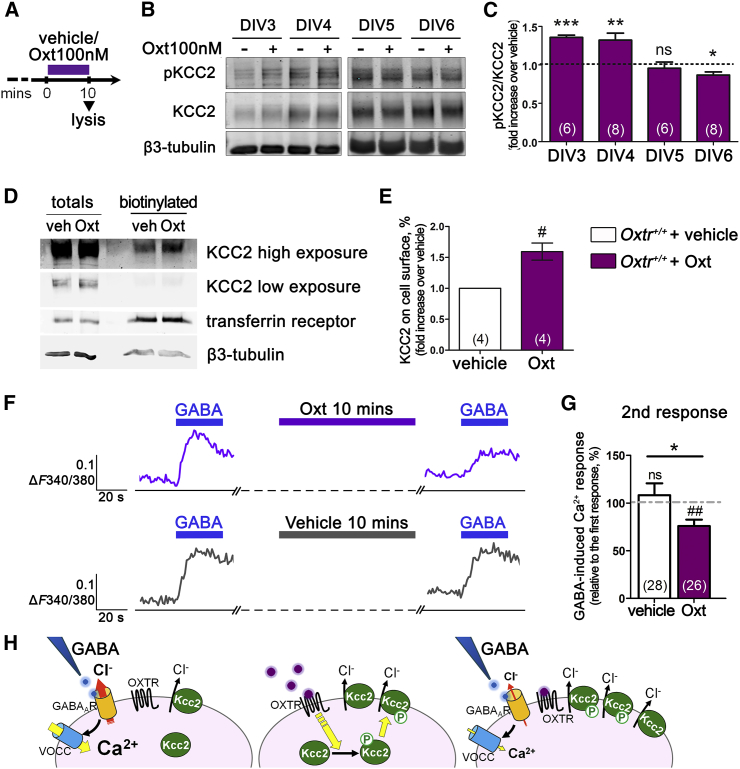
Oxt Increases KCC2 Phosphorylation, Promotes Its Insertion in the Plasma Membrane, and Reduces Excitatory GABA Responses at DIV3 and DIV4, but Not Later (A) Scheme of Oxt administration: 100 nM Oxt (or vehicle) was administered to neurons 10 min before lysis. (B) Representative immunoblots of pKCC2 and total KCC2. β3-tubulin was used as loading control. (C) Quantification of pKCC2/total KCC2 as fold increase over vehicle treated age-matched samples (dotted line). See Figure S2A for *Oxtr* expression during development and Figures S2B–S2E for pKCC2 levels in *Oxtr*^*+/+*^ and *Oxtr*^−/−^ neurons upon Oxt administration. (D) Immunoblot of a representative biotinylation experiment on DIV4 *Oxtr*^*+/+*^ neurons. β3-tubulin and transferrin receptor were used as loading controls for total lysates (15 μg) and biotinylated surface proteins (150 μg). (E) Quantification of biotinylated samples obtained by normalization on respective total lysates and correction for the amounts loaded. Data shown as fold increase over vehicle-treated samples. (F) Representative temporal plots of GABA-induced [Ca^2+^]_i_ changes in an Oxt-treated (purple trace) and a vehicle-(gray trace) neuron. (G) Percent variation in the amplitude (Δ*F*340/380) of GABA-induced responses upon Oxt or vehicle administration, calculated in individual cells. See Figure S2F for KCl-induced responses at the end of Calcium-imaging experiments. (H) Modulation by Oxt of the GABA-induced Ca^2+^ response in *Oxtr*^*+/+*^ neurons at DIV4. From the left: GABA administration induces a Ca^2+^ response via GABA_A_R and VOCC opening; 10-min Oxt treatment increases KCC2 phosphorylation, promotes its membrane insertion/stabilization, and increases Cl^−^ extrusion; a second GABA administration elicits a smaller depolarization and a reduced Ca^2+^ response. Data are presented as mean ± SEM; n numbers in brackets. Student’s t test: ^∗^p < 0.05, ^∗∗^p < 0.01, ^∗∗∗^p < 0.001; one-sample t test: ^#^p < 0.05 and ^##^p < 0.01. All mean, SEM, n, and p values are listed in Table S3.

To test for direct effects of Oxt on KCC2 insertion/stabilization at the plasma membrane, we evaluated the pKCC2/KCC2 ratio in *Oxtr*^*+/+*^ neurons treated with 100 nM Oxt for 10 min (Figure 3A). Oxt significantly increased the pKCC2/KCC2 ratio at DIV3 and DIV4; however, at DIV5, Oxt failed to induce any increase in pKCC2, and a small reduction was observed at DIV6 (Figures 3B and 3C). Oxt-induced action was dose dependent, being Oxt-active down to a concentration of 1 nM (Figures S2B and S2C), and required the presence of Oxtr (Figures S2D and S2E). These findings indicate that in cultured neurons, Oxt, through its receptor, promotes KCC2 phosphorylation only in a very early and restricted time window.

Oxt-promoted membrane insertion of KCC2 was then verified by surface biotinylation experiments on DIV4 neurons. As shown in Figures 3D and 3E, 10-min treatment with 100 nM Oxt increased surface KCC2 by almost 60%.

Finally, to test if an increased membrane KCC2 could alter neuronal responses to GABA, we measured GABA-induced Ca^2+^ transients in *Oxtr*^*+/+*^ neurons before and after Oxt application. Ca^2+^ rises were significantly blunted after Oxt administration (purple trace in Figures 3F and 3G) but not after vehicle treatment (gray trace in Figures 3F, 3G, and S2F). These findings indicate that Oxt is able to reduce GABA-induced depolarization by promoting KCC2 insertion/stabilization at the neuronal plasma membrane (Figure 3H).

### Oxt Phosphorylation of KCC2 at Ser940 Was Mediated by an Oxtr/Gq/PKC-Dependent Pathway

**Figure 4:**
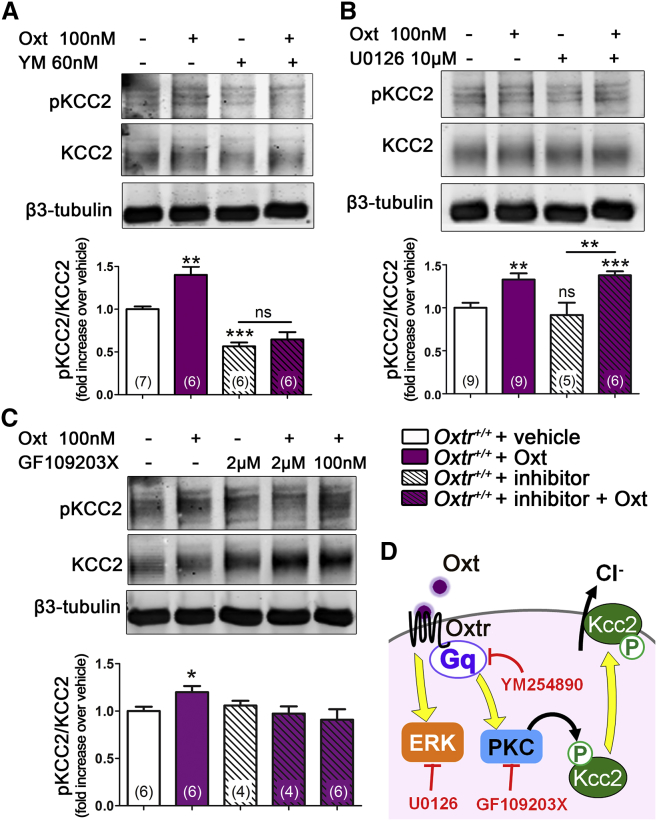
Oxytocin Increases KCC2 Phosphorylation on Ser940 via a Gq- and PKC-Dependent Pathway (A–C) Representative immunoblots and relative quantifications of pKCC2 in neurons treated with vehicle (white bars) or 100 nM Oxt (purple bars) for 10 min, in the absence (plain bars) or presence (striped bars) of selective inhibitors: (A) Gq inhibitor YM254890 (YM, 60 nM, 5-min pretreatment), (B) MEK inhibitor U0126 (10 μM, 30-min pretreatment), and (C) PKC inhibitor GF109203X (GF, 2 μM or 100 nM, 20-min pretreatment). pKCC2 bands intensities were normalized over total KCC2 and displayed as fold change over vehicle. β3-tubulin was used as loading control. See Figure S3 for Oxt-induced ERK phosphorylation and for PMA-induced KCC2 phosphorylation. (D) The cartoon shows the cellular pathway linking the Oxtr to the insertion/stabilization of KCC2 in the plasma membrane and the targets of the inhibitors used. Data are presented as mean ± SEM; n numbers in brackets. Student’s t test: ^∗^p < 0.05, ^∗∗^p < 0.01, and ^∗∗∗^p < 0.001. All mean, SEM, n, and p values are listed in Table S4.

We characterized the signaling pathways involved in the Oxt-dependent KCC2 phosphorylation at Ser940. Pretreatment of DIV4 *Oxtr*^*+/+*^ neurons with the selective Gq-inhibitor YM254890 fully abolished the Oxt-mediated increase in pKCC2, indicating that Gq activation is required for the Oxt-induced effect. Moreover, the administration of YM254890 per se induced a decrease in pKCC2, suggesting that constitutive Gq activation is involved in the modulation of KCC2 phosphorylation at Ser940 (Figure 4A).

Administration of 100 nM Oxt to DIV4 *Oxtr*^*+/+*^ neurons determined a robust phosphorylation of extracellular signal-regulated kinase (ERK), a known downstream target of Oxtr (Rimoldi et al., 2003) (Figures S3A and S3B). Neither basal nor Oxt-induced pKCC2 levels were modified by U0126, an inhibitor of ERK phosphorylation (Figure 4B), indicating that ERK activation is not required for KCC2 phosphorylation at Ser940.

Protein kinase C (PKC), a downstream effector of Gq, has been reported to directly phosphorylate Ser940 and to increase the surface expression of KCC2 (Bos et al., 2013, Kahle et al., 2013, Lee et al., 2007). We confirmed that PKC induces KCC2 phosphorylation at early (DIV4 abd DIV6) and late (DIV11) stages of in vitro development by applying phorbol 12-myristate 13-acetate (PMA), a PKC activator, to *Oxtr*^*+/+*^ neurons (Figures S3C and S3D). To determine if Oxt administration at DIV4 affects KCC2 phosphorylation levels through PKC activation, pKCC2 levels were determined after a pretreatment with the PKC inhibitor GF109203X at 100 nM (to selectively block only the conventional α and β PKC isoforms), and at 2 μM (to inhibit conventional and novel PKC isoforms; Martiny-Baron et al., 1993) (Figure 4C). Both concentrations of GF109203X prevented the Oxt-induced increase in pKCC2, demonstrating that the conventional isoforms of PKC are involved in the Oxtr/Gq-mediated pathway of KCC2 phosphorylation (Figure 4D).

### Mature *Oxtr*^−/−^ Hippocampal Neurons Displayed an Altered E/I Balance

**Figure 5:**
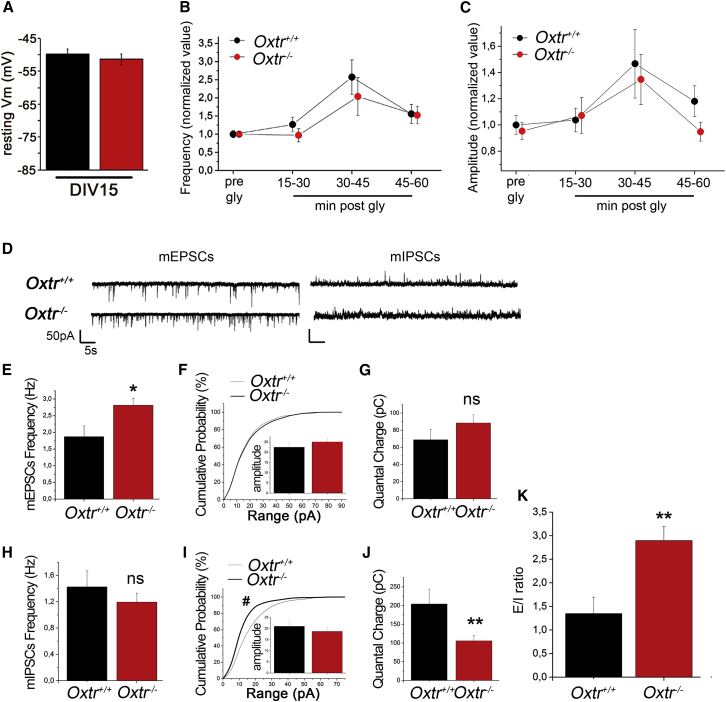
Mature *Oxtr*^−/−^ Neurons Have Normal Resting Vm but Display an Impaired E/I Ratio (A) Measurement of resting membrane potential (Vm) from DIV14 hippocampal neurons did not show any differences between *Oxtr*^*+/+*^ and *Oxtr*^−/−^ groups. (B–K) Electrophysiological characterization of neurons at DIV14. (B) Frequency and (C) amplitude of chemically induced LTP. (D) Representative traces of spontaneous mEPSCs and mIPSCs. Quantification of (E) mEPSC and (H) mIPSCs frequencies. Analysis of (F) mEPSC and (I) mIPSC amplitudes shown as cumulative distribution and mean value (insert). Mean quantal charge transferred during individual (G) mEPSCs and (J) mIPSCs. (K) Excitatory/inhibitory ratio evaluated on the frequencies of miniature events in individual neurons, as described in Supplemental Experimental Procedures. See Figure S4 for dendritic spines analysis. Data are presented as mean ± SEM. Student’s t test: ^∗^p < 0.05 and ^∗∗^p < 0.01; and Kolmogorov-Smirnov test on cumulative distribution: ^#^p < 0.05. All mean, SEM, n, and p values are listed in Table S5.

Due to the relevance of the GABA switch on development and function of neuronal networks, the consequences of the delayed GABA switch observed in *Oxtr*^−/−^ neurons may persist beyond immature stages. To address this issue, we analyzed the morphological and electrophysiological properties of mature *Oxtr*^−/−^ neurons in culture. No differences were found in the number and morphology of dendritic spines (Figure S4) or in resting membrane potential between *Oxtr*^−/−^ and *Oxtr*^*+/+*^ neurons (Figure 5A). Normal functional responses to the chemically induced long-term potentiation (LTP) were observed, indicating normal plasticity of excitatory synapses (Figures 5B and 5C). We then looked for possible alterations of neuronal functions by recording miniature excitatory and inhibitory postsynaptic currents (mEPSCs and mIPSC, respectively; Figure 5D). Measurement of mEPSCs revealed a significant increase in the frequency of excitatory events in *Oxtr*^−/−^ neurons that was not associated with changes in the mean amplitude and quantal charge (Figures 5E–5G). The same analysis performed on inhibitory events revealed no changes in terms of mean frequency but a significant decrease in the amplitude values of *Oxtr*^−/−^ neurons (Figures 5H and 5I) accompanied by a significantly reduced quantal charge (Figure 5J). These results indicate an unbalance between excitation and inhibition in *Oxtr*^−/−^ neurons, which show indeed a much higher E/I ratio, calculated for each individual cell by dividing the frequencies of mEPSCs by those of mIPSCs (Figure 5K).

## Discussion

Our data indicate that, in the absence of *Oxtr*, the developmental upregulation of KCC2 is impaired and the GABA switch is delayed. Alterations in the timing of the GABA switch have been previously reported in *Fmr1*^−/−^ mice, a model of fragile X syndrome, and in rats exposed in utero to valproate (VPA), a model of autism (He et al., 2014, Tyzio et al., 2014). Here, we found an analogous impairment in neurons from the *Oxtr*^−/−^ mouse, a model of autism itself (Sala et al., 2011), in line with the hypothesis that a delayed GABA switch may be a feature shared by several neurodevelopmental disorders. Electrophysiological and behavioral deficits in *Fmr1*^−/−^ and VPA animals have been successfully restored by selective drugs targeting either the Oxt system or the Cl^−^ cotransporters (Eftekhari et al., 2014, Tyzio et al., 2014). However, the link between these two players of the GABA switch remained unsolved.

Here, we show for the first time that the lack of Oxtr in neurons affects specifically KCC2 without impairing NKCC1. Interestingly, both Oxt and KCC2 modulates the GABAergic system. Oxt promotes the rapid formation of inhibitory synapses in adult hypothalamic GABAergic neurons (Theodosis et al., 2006), while KCC2 is fundamental for the maturation of interneurons (Bortone and Polleux, 2009), and its overexpression can increase the density of inhibitory synapses and GABA_A_R clusters (Chudotvorova et al., 2005). We previously reported, in *Oxtr*^−/−^ neurons, an increased ratio between excitatory and inhibitory synapses (Sala et al., 2011), originating from an increase in excitatory synapses and/or a decrease in the inhibitory ones. We report here an increase in the number of functional glutamatergic presynaptic boutons (as suggested by the increased mEPSC frequency) in the presence of unaltered GABA presynaptic input (as suggested by the unchanged mIPSC frequency). An alteration at inhibitory postsynapses in *Oxtr*^−/−^ neurons was revealed by the decreased quantal charge and amplitude of mIPSCs, most likely arising from a change in Cl^−^ homeostasis and GABA_A_ reversal potential. An increased excitatory presynaptic neurotransmission coupled with a reduced postsynaptic inhibition are thus at the basis of the E/I unbalance observed in *Oxtr*^*−/−*^ neurons.

Our present data indicate that KCC2 is one of the molecular players through which the Oxt system could increase the inhibitory tone in hippocampal neurons. However, different effects of Oxt on neurotransmission during development have been observed in different regions; for instance, in the sensory (but not in the prefrontal) cortex, Oxt was found to promote excitatory, rather than inhibitory, activity (Zheng et al., 2014).

Very importantly, a role of Oxt in postnatal brain development is emerging as a relevant novel aspect of this neuropeptide (Grinevich et al., 2014). Indeed, the Oxt system was found to be involved in experience-dependent development of sensory cortices, a process occurring in a restricted critical period of early postnatal life (Zheng et al., 2014).

Within this line of evidence, our finding that Oxt modulates KCC2 expression/stabilization at the plasma membrane only in a very early and narrow time window (by DIV4) is particularly intriguing. The subsequent disappearance of this Oxt effect apparently depends not on a downregulation of its receptor, as real-time qPCR in *Oxtr*^*+/+*^ neurons reveals a developmental upregulation of the Oxtr transcript (up to 8-fold by DIV11; Figure S2A), but most likely on a change in the Oxtr-activated pathway. Analogous age-dependent effects on KCC2 were described for brain-derived neurotrophic factor, being it a positive modulator of KCC2 transcription in immature neurons and a repressor in mature ones (Choe et al., 2015, Ludwig et al., 2011, Rivera et al., 2002).

Oxt actions in early postnatal events seem therefore to result in unique outcomes, which may depend, among other factors, on the specific signaling pathways activated. Oxtr is a promiscuous receptor coupled to several G protein isoforms, triggering multiple signaling cascades (Busnelli et al., 2012). Here, we show that the Gq/PKC pathway in the first stages of development is involved in KCC2 phosphorylation at Ser940, a modification that confers KCC2 with a higher stability at the cell surface, increasing its activity and reducing GABA-induced depolarization. Oxtr-activated pathways may change in the following stages of development inducing different effects in more mature neurons. Elucidating the heterogeneity of Oxt-induced responses is compelling, since this peptide has been proposed as a treatment in a number of neurodevelopmental and neuropsychiatric conditions (Grinevich et al., 2014, Meyer-Lindenberg et al., 2011). Although exogenous Oxt administration to adult autistic patients inconsistently improved symptoms (Young and Barrett, 2015), it proved successful on the onset and progression of neurodevelopmental dysfunctions when applied at birth in animal models of autism and Prader-Willi syndrome (Peñagarikano et al., 2015, Tyzio et al., 2014, Meziane et al., 2015).

The identification of KCC2 as an Oxt target provides a better understanding of the role and therapeutic potential of Oxt for the treatment of neurodevelopmental disorders.

## Experimental Procedures

### Animals and Primary Hippocampal Cultures

*Oxtr*^*+/+*^ and *Oxtr*^−/−^ mice (Takayanagi et al., 2005) were rederived on a C57BL/6 genetic background. All animal procedures were approved by the Italian Ministry of Health (authorization no. 295/2012A; December 20, 2012; protocol no. 1/2014). All experiments were performed in accordance with the Italian legislation. Embryonic day 18 dissociated hippocampal neurons were obtained as described in the Supplemental Experimental Procedures.

### Calcium Imaging and Electrophysiology

For calcium imaging recordings, hippocampal neurons were loaded with Fura-2/AM. [Ca^2+^]_i_ was measured as the fluorescence *F*340/380 ratio. Changes over baseline (Δ*F*340/380) higher than 0.05 units in response to 100 μM GABA were considered depolarizing events.

GABA_A_ reversal potential was calculated from single-channel recordings in cell-attached configuration in DIV8 neuron and corrected for the recorded membrane potential. Whole-cell patch-clamp recordings were performed in DIV14 neurons. mEPSCs and mIPSCs were recorded by holding neurons at the reversal potential for GABAergic (−70 mV) and glutamatergic (+10 mV) responses in a solution containing 1 μM TTX. See the Supplemental Experimental Procedures for details.

### Biochemistry and Molecular Biology

For biotinylation assays, neurons were incubated with ice-cold EZ-Link-Sulfo-NHS-LC-biotin (1 mg/ml), quenched with 50 mM glycine, lysed, and loaded on Streptavidin beads. Biotinylated proteins were separated on SDS-polyacrylamide gels. In western blotting experiments, specific bands were visualized with secondary antibodies conjugated with infrared-emitting fluorophores and signals were quantified using an Odyssey scanner (Li-Cor). For real-time qPCR, cDNA samples were amplified using TaqMan Gene Expression Assay probes. See the Supplemental Experimental Procedures for details.

### Statistics

Statistical analysis was performed with GraphPad Prism5 software. The tests used to assess data significance are indicated in the figure legends. For details, see the Supplemental Experimental Procedures.

## Author Contributions

M.L. designed and conducted cellular, biochemical, and calcium imaging experiments. M.B. contributed to neuronal culture preparation. F.A. and M.M. designed and performed electrophysiological experiments. C.V. supervised the calcium imaging experiments. B.C. conceived and supervised the project. All authors wrote the manuscript and gave final approval for publication.

## Supplemental Information

Document S1. Supplemental Experimental Procedures, Figures S1–S4, and Tables S1–S5

Document S2. Article plus Supplemental Information
