# Supplementary material for: The Timing of the Excitatory-to-Inhibitory GABA Switch Is Regulated by the Oxytocin Receptor via KCC2
Source: Cell Rep. 2016 Mar 24;15(1):96–103. doi: 10.1016/j.celrep.2016.03.013 (PMC4826440; doi:10.1016/j.celrep.2016.03.013)
Supplement: Document S1. Supplemental Experimental Procedures, Figures S1–S4, and Tables S1–S5 [file mmc1.pdf]

Cell Reports, Volume *15*

Supplemental Information

The Timing of the Excitatory-to-Inhibitory GABA Switch Is Regulated by the Oxytocin Receptor via KCC2

Marianna Leonzino, Marta Busnelli, Flavia Antonucci, Claudia Verderio, Michele Mazzanti, and Bice Chini

SUPPLEMENTAL DATA

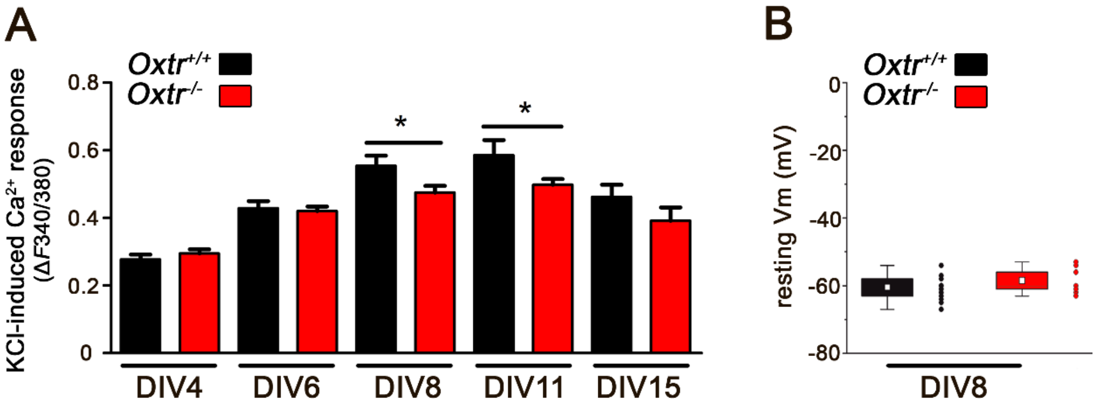

Figure S1 (related to Figure 1). Evaluation of KCl-induced depolarization and resting membrane potential in *Oxtr*$^{+/+}$ and *Oxtr*$^{-/-}$ hippocampal neurons.

(A) The amplitude of high K^+-induced Ca^{2+} responses, indicative of VOCC expression, was measured with Ca^{2+} imaging at DIV4, 6, 8, 11 and 15 and expressed as ΔF340/380. More than 30 neurons from at least 2 different preparations were analyzed per timepoint.

(B) Average resting membrane potential (Vm) of *Oxtr*$^{+/+}$ (n= 15) and *Oxtr*$^{-/-}$ (n= 9) single neurons.

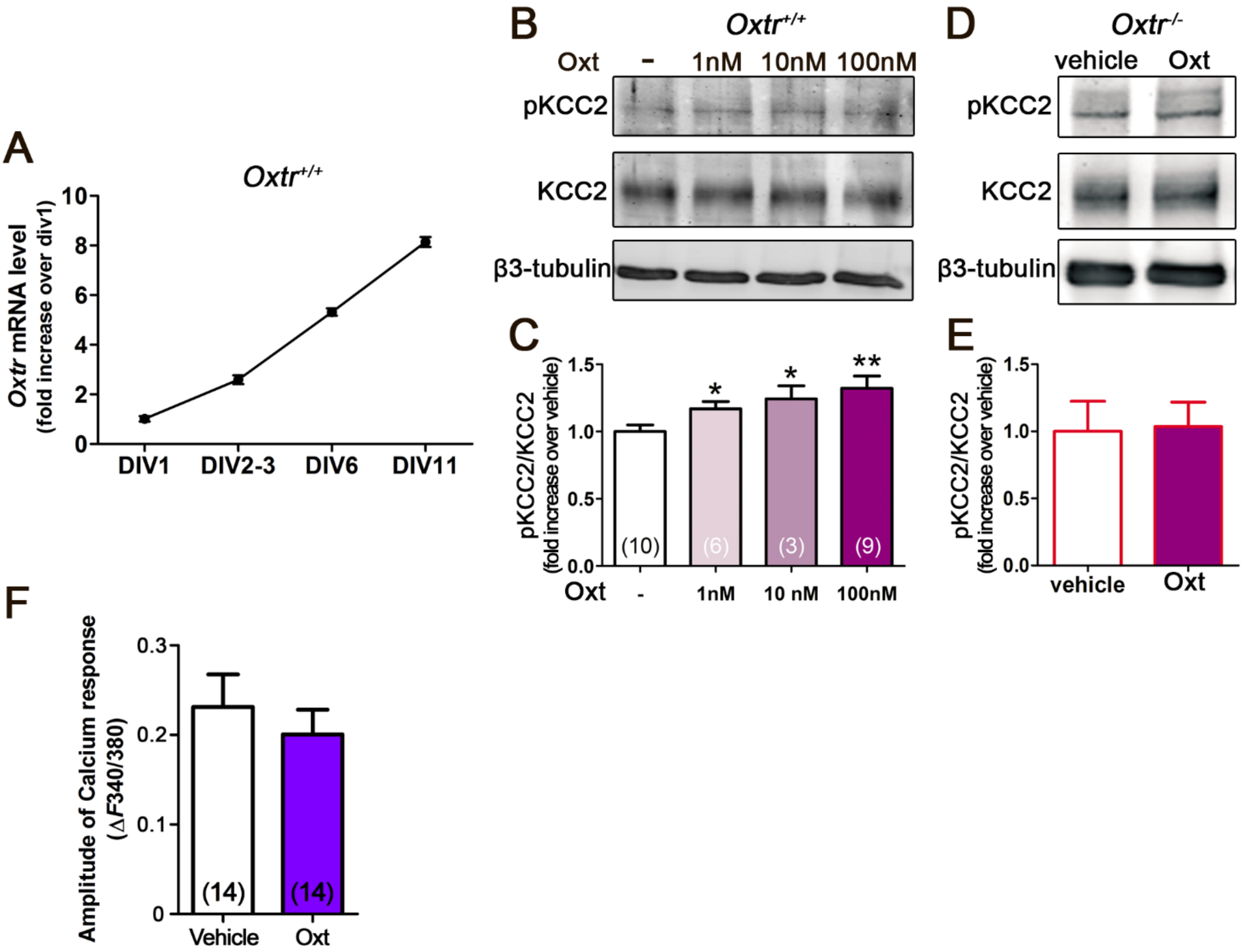

Figure S2 (related to Figure 3). Oxt modulates pKCC2 levels through the Oxytocin receptor in a dose-dependent way.

(A) Oxtr expression was detected in *Oxtr*$^{+/+}$ neurons already at DIV1 and increased over time. Relative levels of the transcript determined by qRT-PCR are shown as fold change over DIV1 (N=3). **(B-C)** Higher Oxt dose administered to DIV4 *Oxtr*$^{-+/+}$ neurons (10 min treatment) increased relative KCC2 phosphorylation. **(B)** Representative immunoblot and **(C)** quantification of pKCC2/total KCC2 shown as fold increase over vehicle treated age-matched samples. β3-tubulin was used as loading control. N numbers in brackets. **(D-E)** 10 min Oxt treatment (100nM) at DIV4 has no effects on *Oxtr*$^{-/-}$ neurons. **(D)** Representative immunoblot and **(E)** relative quantification of pKCC2/total KCC2 shown as fold increase over vehicle treated age-matched samples. β3-tubulin was used as loading control. (N=3). **(F)** Vehicle- and Oxt-treated neurons showed similar KCl-induced responses at the end of Calcium-imaging experiments, indicating that they both maintained a good neuronal viability. N numbers in brackets. Data are presented as mean ± SEM; Student *t* test: *p<0.05; **p<0.01.

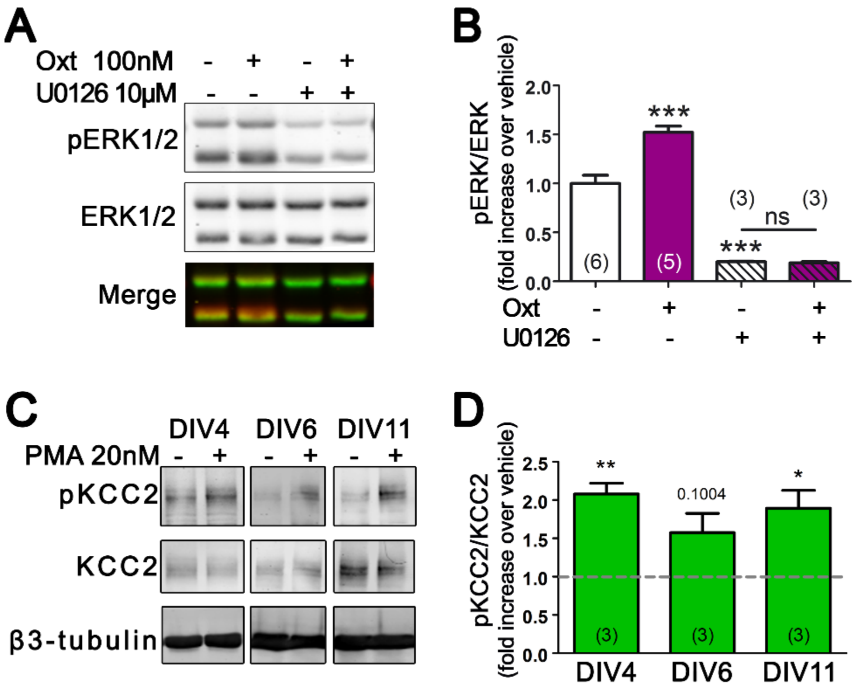

Figure S3 (related to Figure 4). Oxt-mediated signaling during development.

(A-B) Oxt treatment (100nM, 10 min) induces ERK1/2 phosphorylation in *Oxtr*$^{+/+}$ neurons at DIV4. **(A)** Immunoblot of phosphorylated and total ERK1/2 in *Oxtr*$^{+/+}$ neurons at DIV4 upon 10 min treatment with 100nM OXT (purple bars) in the absence (plain bars) or presence (patterned bars) of the MEK inhibitor U0126 (10µM, 30 min pretreatment). The signals coming from both IR-channels are shown in the "Merge" panel (red, p-ERK1/2-GaM680; green, total ERK1/2-GaR800). **(B)** Quantification of ERK1/2 phosphorylation calculated as the ratio between p-ERK1/2 and total ERK1/2 and displayed as fold change over vehicle-treated samples. **(C-D)** Exogenous activation of PKC with PMA (20nM, 30 min) increased KCC2 phosphorylation at DIV4 (*t*-test, p=0.056), DIV6 (even though not statistically significant; *t*-test, p=0.1004) and DIV11 (*t*-test, p=0.0219) indicating that KCC2 remains a target of PKC-mediated phosphorylation during in vitro development. **(C)** Immunoblot of phosphorylated and total KCC2 in *Oxtr*$^{+/+}$ neurons at DIV4, DIV6 and DIV11 treated with vehicle or PMA. **(D)** Quantification of KCC2 phosphorylation calculated as the ratio between p-KCC2 and total KCC2 and displayed as fold change over age-matched vehicle-treated samples (dotted line). Data presented as mean ± SEM; Student *t* test: $*p<0.05$; $**p<0.01$; $***p<0.001$; N numbers in brackets.

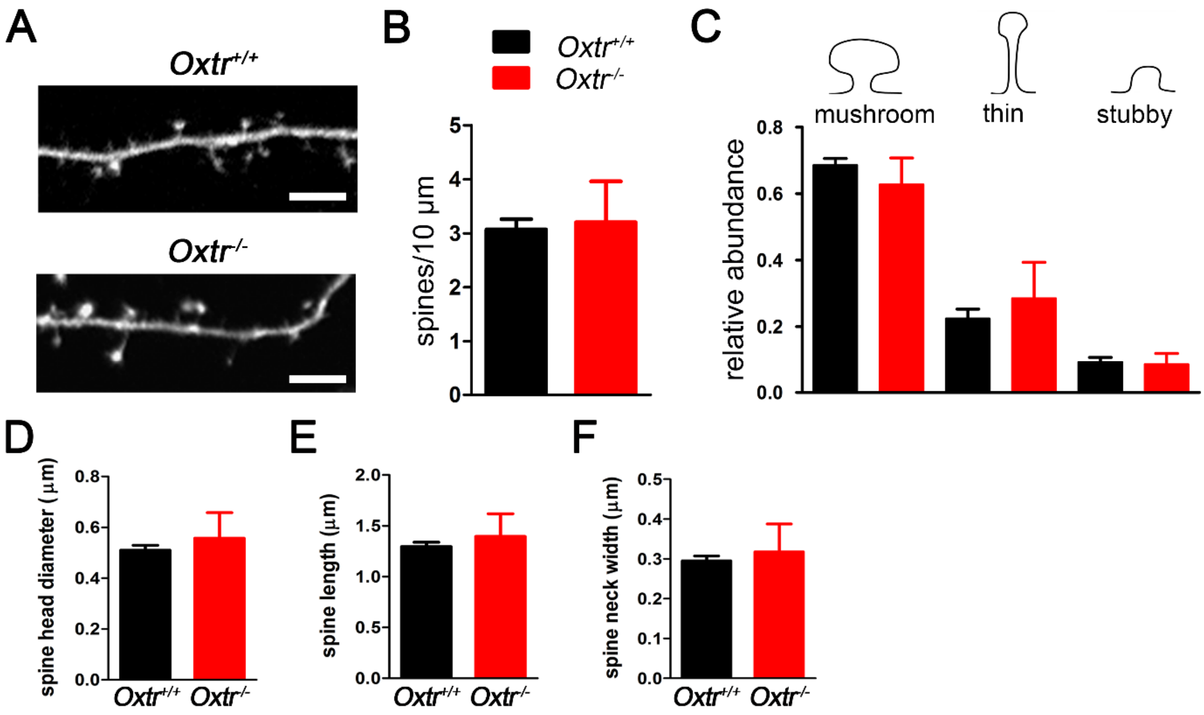

Figure S4 (related to Figure 5): Mature hippocampal neurons from *Oxtr*$^{+/+}$ and *Oxtr*$^{-/-}$ mice have similar spine density and morphology.

(A) Analysis of spine density and morphology at DIV17 in GFP-transfected neurons reveals no difference between *Oxtr*$^{+/+}$ and *Oxtr*$^{-/-}$ cells. Scale bars 10 µm. **(B)** The spine density was calculated as number of spine per 10µm of dendrite. **(C)** The abundance of different types of spines (mushroom, thin and stubby) was analyzed by classifying spines in categories based on morphological parameters: **(D)** spine head diameter, **(E)** spine length and **(F)** spine neck width. Data presented as mean ± SEM. Student *t* test: $p>0.05$. 5-6 neurons analyzed for each genotype.

Table S1. Mean values, SEM, number of samples and p values of data displayed in Figure 1

**Calcium imaging**	*Oxtr*$^{+/+}$			*Oxtr*$^{-/-}$			p value<br>Bonferroni *post hoc*<br>* Student *t*-test<br># One-sample *t*-test<br>& Student *t*-test with Welch's correction
	mean	SEM	N	mean	SEM	N	
Figure 1C – GABA-responsive cells							
DIV4	71.7%	5.7	10	86.7%	3.6	8	>0.05
DIV6	32.6%	7.5	9	59.8%	5.2	8	<0.001
DIV8	8.5%	1.8	9	37.7%	5.3	6	<0.001
DIV11	0		5	14.3%	4.0	10	# 0.0051
DIV15	0		5	0		6	-
Figure 1D - ΔGABA							
DIV4	122.7	5.921	226	143.4	6.354	249	* 0.0182
DIV6	109.6	9.732	80	131.8	7.255	175	* 0.0691
DIV8	70.4	5.883	19	92.59	7.819	75	* 0.0261
DIV11				73.62	5.079	41	# <0.0001
**Electrophysiology – GABA reversal potential**							
Figure 1G	-60.71	0.665	14	-49.5	2.232	22	& <0.0001

Table S2. Mean values, SEM, number of samples and p values of data displayed in Figure 2

	Oxtr$^{+/+}$			*Oxtr*$^{-/-}$			p value Bonferroni *post hoc*<br>* Student *t*-test<br># One-sample *t*-test
	mean	SEM	N	mean	SEM	N	
**Real Time**							
Figure 2A – NKCC1							
DIV2	0.4866	0.0352	3	0.2664	0.0193	2	>0.05
DIV6	0.25	0.0378	3	0.235	0.125	2	>0.05
DIV11	0.4066	0.1481	3	0.24	0.0208	3	>0.05
Figure 2B – KCC2							
DIV2	2.35	0.3871	4	1.09	0.3927	3	>0.05
DIV6	15.5625	6.6892	4	2.7375	1.1677	4	>0.05
DIV11	34.6325	12.2596	4	4.835	1.5706	4	<0.01
**Western Blot**							
Figure 2D							
DIV3 (inset)			12	1.16	0.1123	12	# 0.1824
DIV6	7.5859	1.4620	6	5.2268	0.7135	6	> 0.05
DIV11	17.2715	2.1280	6	10.4111	1.4670	5	<0.01
Figure 2E							
DIV3 (inset)			12	1.125	0.1501	12	# 0.4216
DIV6	1.5841	0.1283	9	0.9050	0.1585	9	>0.05
DIV11	2.3746	0.6405	6	1.0026	0.2271	9	<0.001
Figure 2G							
PN0	1.000	0.1880	2	1.132	0.1920	3	* 0.6753
PN6	12.76	1.963	3	5.575	1.488	3	* 0.0434
PN60	27.56	4.502	3	11.55	0.3355	3	* 0.0239

Table S3. Mean values, SEM, number of samples and p values of data displayed in Figure 3

	mean	SEM	N	p value<br>Student *t*-test<br># One-sample t-test
**WB- Oxt treatment - ** Figure 3C				
DIV3	1.357	0.02986	6	<0.0001
DIV4	1.321	0.09216	8	0.0086
DIV5	0.9556	0.08156	6	0.7518
DIV6	0.8672	0.041	8	0.0418
**Biotinylation Assay - ** Figure 3E				
Oxt	1.594	0.1379	4	# 0.0231
**Calcium imaging -** Figure 3G				
Oxtr$^{+/+}$	108.2	12.53	28	# 0.5173
Oxtr-/-	75.92	6.652	26	# 0.0013<br>0.0282

Table S4. Mean values, SEM, number of samples and p values of data displayed in Figure 4

**Western Blot**	Oxt			Inhibitor			Inhibitor+Oxt			
	mean	SEM	p value[a]	mean	SEM	p value[a]	mean	SEM	p value[a]	p (over inhibitor)[a]
Figure 4A - YM	1.402	0.0954	0.0072	0.5655	0.0450	<0.0001	0.6457	0.0857	0.0017	0.4272
Figure 4B - U0126	1.328	0.0720	0.0027	0.916	0.1432	0.5317	1.379	0.0461	0.0005	0.0088
Figure 4C - GF 2μM	1.199	0.0653	0.0376	1.058	0.0503	0.4513	0.908	0.1116	0.768	0.4636
Figure 4C - GF100nM							0.973	0.0762	0.4005	0.2888

[a] Student *t*-test

Table S5. Mean values, SEM, number of samples and p values of data displayed in Figure 5

	Oxtr$^{+/+}$ (*mean±SEM, N)*	*Oxtr*$^{-/-}$ (*mean±SEM,* N)	p value Student *t*-test
**Electrophysiology – Resting membrane potential**			
Figure 5A	-49.78±1.556 N=8	-51.33±1.641 N=9	0.4975
**Electrophysiology – Chemical LTP**			
Figure 5B - frequency			
pre-gly	1±0,09 N=16	1±0.07 N=15	1-way ANOVA Dunn's Method*:* *Oxtr*$^{+/+}$=0.004 *Oxtr*$^{-/-}$=0.025
15 post	1,26±0,20 N=8	0.97±0.18 N=8	
30 post	2,57±0.47(**) N=10	2.03±0.52 (*) N=7	
45 post	1.55±0.26 N=8	1.52±0.23 N=9	
Figure 5C – amplitude			
pre-gly	1±0.071 N=16	0.95±0.06 N=15	1-way ANOVA Holm-Sidak method: *Oxtr*$^{+/+}$=0.026 *Oxtr*$^{-/-}$=0.033
15 post	1.037±0.09 N=8	1.07±0.13 N=8	
30 post	1.46±0.26(*) N=10	1.34±0.19(*) N=7	
45 post	1.18±0.11 N=8	0.94±0.07 N=9	
**Electrophysiology – mEPSCs**			
Figure 5E (frequency)	1.871±0.32 N=9	2.776±0.24 N=15	0.037
Figure 5F (mean amplitude) (cumulative distribution)	22.48±2.24 N=9	25.08±1.62 N=15	0.349 *KS* test: p=0.22
Figure 5G (quantal charge)	68.72±12.02 N=9	88.45±9.71 N=15	0.107
**Electrophysiology - mIPSCs**			
Figure 5H (frequency)	1.42±0.24 N=9	1.19±0.13 N=15	0.202
Figure 5I (mean amplitude) (cumulative distribution)	21±1.99 N=9	18.69±2.10 N=14	0.013 *KS* test: p=0.004
Figure 5J (quantal charge)	204.11±39.80 N=9	93.05±17.68 N=14	0.022
**Electrophysiology – E/I ratio**			
Figure 5K	1.34±035 N=8	3.49±0.54 N=14	0.006

SUPPLEMENTAL EXPERIMENTAL PROCEDURES

Animals

Oxtr$^{+/+}$ and *Oxtr*$^{-/-}$ mice, rederived on a C57BL/6 genetic background (Charles Rivers, Calco, Italy), were stabulated in standard conditions, with *ad-libitum* access to food and water. Colony propagation has been carried out by heterozygous mating and litters were genotyped by PCR. Every animal procedure used was in strict accordance with standard ethical guidelines (European Community Guidelines on the Care and Use of Laboratory Animals 2010/63/EU) and the Italian legislation on animal experimentation (D.Lvo 116/92).

Primary hippocampal cultures

Embryonic day 18 dissociated hippocampal neurons were obtained from *Oxtr*$^{+/+}$ and *Oxtr*$^{-/-}$ timed pregnant mice as described by Kaech and Banker (Kaech and Banker, 2006), with slight modifications. Briefly, tissue dissociation was carried out with an enzymatic treatment (0.25% trypsin for 20 min at 37°C) followed by mechanic dissociation with a fire-smoothed Pasteur pipette. Dissociated cells were plated (30 000 cells/cm^2) in poly-L-lysine coated multiwell dishes containing Neurobasal medium (Life Technologies) additioned with B27 supplement (2% v/v; Life Technologies), L-glutamine (2mM), penicillin/streptomycin (100U/ml) and 25µM Glutamate. Five hours after plating the medium was replaced with a glutamate-free one to avoid excitotoxicity. Neurons were then maintained at 37°C in humidified atmosphere (95% air and 5% CO_2), and half of the medium was refreshed once a week.

Electrophysiology

Patch electrodes (GB150F-8P with filament, Science Products) are pulled from hard borosilicate glass on a Brown-Flaming P-87 puller (Sutter Instruments, Novato, CA, USA) and fire polished to a tip diameter of 1-1.5 µM and an electrical resistance of 4-6 MΩ.

Excitatory and inhibitory currents have been analyzed in DIV14 hippocampal neurons by whole-cell patch-clamp recordings of EPSCs and IPSCs in miniature using a Multiclamp 700A amplifier (Molecular Devices) and pClamp-10 software (Axon Instruments, Foster City, CA). Recordings were carried out in voltage-clamp mode in the presence of tetrodotoxin (TTX, 1 µM). Currents were sampled at 5 kHz, filtered at 2-5 kHz and analyzed (off-line) with Clampfit-pClamp 10.2 software. For the evaluation of the E/I balance, mEPSCs or mIPSCs were recorded using an internal solution containing CsGluconate (130mM), CsCl (8mM), NaCl (2mM), HEPES (10mM), EGTA (4mM), MgATP (4mM) and Tris-GTP (0.3mM), pH 7.4. The external solution (Krebs-Ringer's-HEPES - KRH) contained NaCl (125mM), KCl (5mM), $MgSO_4$ (1.2mM), KH_2PO_4 (1.2mM), $CaCl_2$ (2mM), glucose (6mM) and HEPES-NaOH (25mM), pH 7.4. The E/I ratio was calculated by dividing mEPSCs and mIPSCs frequencies measured in the same neuron. Thresholds were set at 8pA for mEPSCs and at 6pA for mIPSCs.

For the chemical LTP experiment (Fossati et al., 2015; Menna et al., 2013), basal excitatory events were monitored using an intracellular solution of KRH containing TTX (0.5µM), bicuculline (20µM, Tocris) and strychnine (1µM, Sigma-Aldrich). Glycine (100µM, Sigma-Aldrich) was subsequently applied for 3 min at room temperature in Mg^{2+}-free KRH containing TTX, bicuculline and strychnine and potentiation of EPSCs currents was recorded from 15 to 60 min after glycine delivery.

For cell-attached experiments the patch pipette was filled with the bath solution (in mM: 140 NaCl, 0.5 $MgCl_2$, 10 HEPES, 0.1 $CaCl_2$, 1 EGTA, pH 7.4) with the addition of 5 mM TEA and 100 □M DIDS to minimized K^+ and CLC ionic channel activity. Patch pipette tip was dipped in the described solution and back filled with the same solution supplemented with 10 □M GABA immediately before the experiment. Patches showing channel activity in the first 1-2 min after reaching the tight seal cell attached mode were discharged. Channel activity was observed using a step protocol from -80 to +80 mV pipette voltage in 20 mV steps and 400 ms duration. Routinely after 15 min of current recordings the membrane patch was broken in current-clamp mode to monitor the cell resting potential. Although the electrode contained an inappropriate solution for whole cell experiments, the measured resting potential, obtained at the end of each cell attached experiment, matched the values obtained using gramicidin perforated patch or with an electrode filled with the intracellular-like solution for whole cell recording (K-aspartate, 120mM; NaCl, 10mM; EGTA, 10mM; $MgCl_2$, 2mM; $CaCl_2$, 4mM; MgATP, 3mM; Na_2 GTP, 0.2mM; HEPES–KOH, 10mM, pH 7.2.). The amplitude of GABA-induced $GABA_A$ receptor channel was measured at different test potentials and plotted on a current/voltage relationship after the voltage was adjusted according to the measured membrane potential. Linear fit of the experimental points indicate the current reversal potential and in the case of $GABA_A$ receptor, the chloride reversal potential.

Treatments

Oxytocin (Oxt) was purchased from Bachem, GABA and PMA from Sigma-Aldrich. The selective Gq inhibitor YM-254890 (Takasaki et al., 2004) was a generous gift of Jun Takasaki (Astellas Pharma Inc., Tsukuba, Japan), whereas the MEK inhibitor U0126 was purchased from Cell Signalling and the PKC inhibitor GF109203X from TOCRIS. All drugs were pre-diluted in Neurobasal or KRH media and then applied to neurons at the final concentration and at the indicated time.

Calcium imaging

Hippocampal neurons were loaded with the membrane-permeable fluorescent Ca^{2+} indicator Fura-2/AM (1 μM; Sigma-Aldrich) for 30 min at 37°C, 5% CO_2. Following dye-loading, the cells were thoroughly washed with KRH buffer, subsequently used as extracellular recording solution. From DIV6 on, 1 μM TTX was added to this extracellular recording solution. Neurons were placed into the recording chamber of an inverted microscope (Axiovert 100, Zeiss) and imaged through a 40x objective (Zeiss).

Fura-2/AM was excited at 380 nm and at 340 nm through a Polychrom V, (TILL Photonics GmbH) controlled by the TillVisION software 4.01. Emitted light was acquired at 505nm at 1Hz, and images collected with a CCD Imago-QE camera (TILL Photonics GmbH).

The fluorescence ratio *F*340/380 was used to express Ca^{2+} concentrations. This parameter was recorded in regions of interest (ROIs) corresponding to neuronal cell bodies, and analyzed along sequential images to follow temporal changes.

After a period of basal recordings, GABA was administered at 100μM. Increases in *F*340/380 ratio (Δ*F*340/380) higher than 0.05 units within 15s after drug administration were considered reliable Ca^{2+} responses. After GABA recordings, neurons were washed with KRH and let to recover for a few minutes, before administering KCl (50mM) to identify viable neurons. Neurons responding to depolarization delivery with a Δ*F*340/380 smaller than 0.08 units were excluded from the analysis.

For the study of Oxt's action, a first GABA administration was followed by a short (2 min) recovery time. Subsequently, 100nM Oxt (or vehicle) was administered and left in the bath for 10 min, then a second GABA challenge was delivered to neurons. The percentage change in the Δ*F*340/380 of the second response with respect to the first one was scored for each individual neuron recorded.

GFP-transfection, imaging and morphological analysis

$Oxtr^{+/+}$ and $Oxtr^{-/-}$ neurons were seeded on glass coverslips at 25,000 cells/cm^2, and transfected at DIV7 with a GFP-expressing vector using Lipofectamine2000 (Life Technologies). For each well of a 12-well dish, 2.5 μg of DNA were used. Neurons were maintained until DIV17, then fixed with 4% paraformaldheyde-4% sucrose (w/v) and imaged with a 63x objective using a 510LSM Meta laser scanning confocal microscope (Zeiss). Focal planes were stacked together in a projection, then dendritic spines were counted manually. For each genotype 5-6 neurons were analyzed. Dendritic length and spine morphology parameters: Spine length (L), head diameter (H), and neck width (N) were measured using the ImageJ software (imagej.nhi.gov/ij/), then spines were assigned to different subclasses according to NeuronStudio software criteria: Mushroom spines: H/N>1.1μm and H>0.35 μm; Thin spines: H/N>1.1μm and H<0.35 μm or H/N<1.1μm and L/H>2.5 μm; Stubby spines: H/N<1.1μm and L/H<2.5 μm.

RNA extraction, cDNA synthesis, and Real-Time PCR

Neurons' RNA content was isolated using the Nucleospin RNA kit (Macherey-Nagel), following manufacturer's instructions. For each sample 500 μg of total RNA were retrotranscribed using the SuperScript III Reverse Transcriptase Kit (Life Technologies) according to manufacturer's instructions. For quantitative Real-Time PCR cDNA samples were amplified in triplicate, using 20 ng per replicate, with appropriate Taqman® Gene Expression Assay' s probes (KCC2: Slc12a5 #Mm00803929_m1; NKCC1: Slc12a2 #Mm00436554_m1; Oxtr: #Mm01182684_m1; Life Technologies). The housekeeping hypoxantine phosphoribosyltransferase 1 (HPRT-1) gene was used as reference gene and amplified in parallel using its specific TaqMan® Assay (Hprt1 #Mm00446968_m1; Life Technologies). Real-Time PCR was performed using the ABI Prims™ 7000 Sequence Detection System (Applied Biosystems). Results were elaborated with the ABI Prism1.2.3 software (Applied Biosystems) using the $2^{-\Delta\Delta Ct}$ method. Target genes (Kcc2, Nkcc1 and Oxtr) were normalized on the reference gene (Hprt-1) and compared with the calibrator (DIV1, set to 1) for each experiment.

Surface biotinylation assay

Neurons at DIV4 were transferred to KRH buffer for 10 min, then Oxt (final concentration 100nM), or KRH were incubated for subsequent 10 min. Treatments were stopped with ice-cold KRH, after which neurons were incubated for 15 min at 6°C with KRH containing EZ-Link-Sulfo-NHS-LC-biotin (1mg/ml). Biotin excess was quenched with two 10-min washes with a 50mM Glycine-KRH buffer solution (adjusted to maintain physiological osmolarity). Neurons were lysed in ice-cold RIPA buffer (NaCl 150mM, Tris-HCl 50mM, pH 7.4, EDTA 1mM, Triton X-100 1% and NP-40 1%) containing proteases and phosphatases inhibitors (Roche), and protein content was assessed by a DC protein assay (Bio-Rad). Equal amounts of proteins were loaded on Streptavidin beads (Ultralink Streptavidin Resin, Pierce) and left to rotate on a wheel for 16 hours. After a brief centrifugation, supernatants containing the non-biotinylated proteins were removed, beads were washed three times with PBS and finally biotinylated proteins were collected by adding 3x-Laemli Buffer (Tris-HCl 375mM, pH 6.8, glycerol 20% w/v, SDS 9% w/v, β-mercaptoethanol 10% v/v and Bromophenol Blue 0.05% w/v) to the beads and heating at 95°C for 3 min. Proteins were then separated by SDS-PAGE and processed for western blotting as described below.

Western blotting

Neurons were lysed in ice-cold RIPA buffer with a Protease Inhibitor Cocktail (Sigma) and Phosphatase Inhibitors (Roche). Protein concentration was determined with the DC protein assay kit (Bio-Rad) and samples were diluted in 3x-Laemli buffer. For Western blotting, 2-4 neuronal preparations were used and in each experiment neuronal samples derived from three independent wells were run for each data point.

Protein samples were separated by SDS-PAGE and then transferred onto a nitrocellullose membrane (GE Healthcare). Unspecific binding sites were saturated by incubation with BSA (5% w/v) for 1 hour at 37°C. Proteins of interest were probed with the proper primary antibody and revealed by secondary antibodies conjugated with infrared-emitting-fluorophores. Signals were detected and quantified using an Odissey scanner (Li-Cor) controlled by the ImageStudio software (Li-Cor). All the antibodies used and the respective suppliers and working dilutions are listed in the table below.

Protein MW (kDa)	Primary Antibody	Secondary Antibody
140	Rabbit Anti-Kcc2 (gift from *C. Rivera*) 1:3000	IRDye® 800CW Goat Anti-Rabbit (*Li-Cor*) 1:2500
140	Rabbit Anti-p-Ser940Kcc2 (*Rockland*) 1:1000	
42-44	Rabbit Anti-ERK1/2 (*Cell Signalling*) 1:1000	
42-44	Mouse Anti-p-ERK1/2 (*Cell Signalling*) 1:1000	IRDye® 680RD Goat Anti-Mouse (*Li-Cor*) 1:5000
55	Mouse Anti-β3-tubulin (*Promega*) 1:1000	
95	Mouse Anti-Transferrin Receptor (*Invitrogen*) 1:1000	

Primary and Secondary antibodies used for western blotting analysis. The molecular weight (MW) of expected bands is reported in the first column. Antibody suppliers are indicated in brackets. Working dilutions of each antibody is also reported.

Statistical analysis

For electrophysiology and Ca^{2+} imaging experiments, data were obtained from at least three different neuronal preparations. For qRT-PCR and Western blotting, replicates consisted in different wells coming from 2-4 independent neuronal preparations. Statistical analysis was performed with GraphPad Prism 5.0 (GraphPad Software, Inc.).

For experiments where *Oxtr*$^{+/+}$ and *Oxtr*$^{-/-}$ were compared repeatedly over time, 2-way ANOVA was used, with "time" and "genotype" as variables, followed by Bonferroni's *post hoc* test. For direct comparisons of two data sets, Student's *t*-test was used, whereas One-sample *t*-test was performed to assess significant differences of datasets from a fixed

control value (i.e. 1.00 or 100%). LTP experiments were analyzed by parametric or non parametric 1-way ANOVA followed by Holm-Sidak or Dunn's *post hoc* tests, respectively. To compare cumulative distributions Kolmogorov-Smirnov (*KS*) test was used. For all the experiments, results were deemed statistically significant when $p<0.05$.

SUPPLEMENTAL REFERENCES

Fossati, G., Morini, R., Corradini, I., Antonucci, F., Trepte, P., Edry, E., Sharma, V., Papale, A., Pozzi, D., Defilippi, P., *et al.* (2015). Reduced SNAP-25 increases PSD-95 mobility and impairs spine morphogenesis. Cell Death Differ *22*, 1425-1436.

Kaech, S., and Banker, G. (2006). Culturing hippocampal neurons. Nature protocols *1*, 2406-2415.

Menna, E., Zambetti, S., Morini, R., Donzelli, A., Disanza, A., Calvigioni, D., Braida, D., Nicolini, C., Orlando, M., Fossati, G., *et al.* (2013). Eps8 controls dendritic spine density and synaptic plasticity through its actin-capping activity. EMBO J *32*, 1730-1744.

Takasaki, J., Saito, T., Taniguchi, M., Kawasaki, T., Moritani, Y., Hayashi, K., and Kobori, M. (2004). A novel Galphaq/11-selective inhibitor. J Biol Chem *279*, 47438-47445.
