# Supplementary material for: The Timing of the Excitatory-to-Inhibitory GABA Switch Is Regulated by the Oxytocin Receptor via KCC2
Source: Cell Rep. 2016 Mar 24;15(1):96–103. doi: 10.1016/j.celrep.2016.03.013 (PMC4826440; doi:10.1016/j.celrep.2016.03.013)
Supplement: Document S2. Article plus Supplemental Information [file mmc2.pdf]

Cell Reports

Report

The Timing of the Excitatory-to-Inhibitory GABA Switch Is Regulated by the Oxytocin Receptor via KCC2

Graphical Abstract

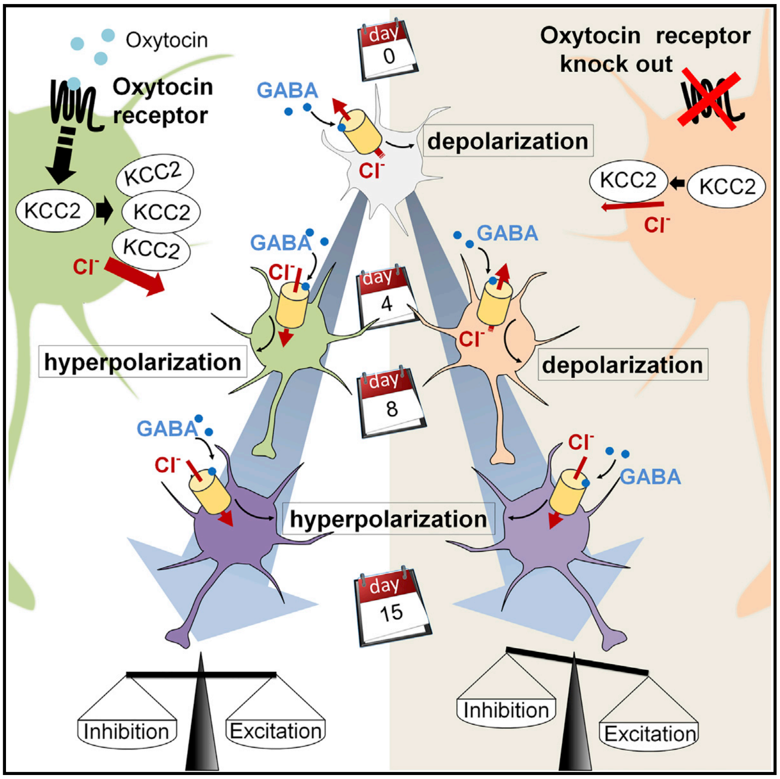

Highlights

- Oxtr is necessary for the correct timing of the GABA switch in developing neurons
- Oxtr modulates the GABA switch by directly regulating the Cl^- transporter KCC2
- OXT actions on KCC2 are restricted to an early and narrow time window
- Oxtr deficit causes long-lasting defects in excitation/inhibition balance in neurons

Authors

Marianna Leonzino, Marta Busnelli, Flavia Antonucci, Claudia Verderio, Michele Mazzanti, Bice Chini

Correspondence

b.chini@in.cnr.it

In Brief

Dysfunctions of the oxytocin system can contribute to neurodevelopmental disorders. Leonzino et al. find that in the absence of the oxytocin receptor, the chloride transporter KCC2 is downregulated, causing a delayed excitatory-to-inhibitory GABA switch and long-lasting electrophysiological alterations in neurons.

CrossMark

Leonzino et al., 2016, Cell Reports *15*, 96–103
April 5, 2016 ©2016 The Authors
http://dx.doi.org/10.1016/j.celrep.2016.03.013

CellPress

OPEN ACCESS
CellPress

Cell Reports
Report

The Timing of the Excitatory-to-Inhibitory GABA Switch Is Regulated by the Oxytocin Receptor via KCC2

Marianna Leonzino,[1,2] Marta Busnelli,[1] Flavia Antonucci,[2] Claudia Verderio,[1,3] Michele Mazzanti,[4] and Bice Chini[1,3,*]
[1]Institute of Neuroscience, Consiglio Nazionale delle Ricerche, Milan 20129, Italy
[2]Department of Biotechnology and Translational Medicine, University of Milan, Milan 20129, Italy
[3]Humanitas Clinical and Research Center, IRCCS, Rozzano, Milan 20089, Italy
[4]Department of Bioscience, University of Milan, Milan 20133, Italy
*Correspondence: b.chini@in.cnr.it
http://dx.doi.org/10.1016/j.celrep.2016.03.013

SUMMARY

Oxytocin and its receptor (Oxtr) play a crucial role in the postnatal transition of neuronal GABA neurotransmission from excitatory to inhibitory, a developmental process known as the GABA switch. Using hippocampal neurons from Oxtr-null mice, we show that (1) Oxtr is necessary for the correct timing of the GABA switch by upregulating activity of the chloride cotransporter KCC2, (2) Oxtr, in a very early and narrow time window, directly modulates the functional activity of KCC2 by promoting its phosphorylation and insertion/stabilization at the neuronal surface, and (3) in the absence of Oxtr, electrophysiological alterations are recorded in mature neurons, a finding consistent with a reduced level of KCC2 and increased susceptibility to seizures observed in adult Oxtr-null mice. These data identify KCC2 as a key target of oxytocin in postnatal events that may be linked to pathogenesis of neurodevelopmental disorders.

INTRODUCTION

To correctly shape neuronal circuits, postnatal brain development requires a finely tuned balance between excitation and inhibition (E/I). Impairments of this balance have been proposed to underlie many neurodevelopmental brain disorders including autism. The most critical determinants of this balance are glutamate and γ-aminobutyric acid (GABA), respectively the main excitatory and inhibitory neurotransmitters. At early stages of development, however, activation of $GABA_A$ receptors ($GABA_AR$) generates membrane depolarization and thus excitation. Therefore, in immature neurons, both glutamate and GABA, by inducing depolarization and Ca^{2+} influx through voltage-operated Ca^{2+} channels (VOCC), work in synergy on proliferation, migration, maturation, and differentiation. As a consequence, the proper timing of GABA transition from depolarizing to hyperpolarizing is fundamental for a correct development of the brain (Ben-Ari et al., 1989).

The switch in GABA polarity has been shown to have a biphasic time course: the first phase is an abrupt and fully reversed switch that is temporally restricted to the delivery period (Tyzio et al., 2006); the second, most commonly referred to as "GABA switch," is a progressive and permanent switch that starts soon after birth and is complete, in rodents, by the end of the first postnatal week (Valeeva et al., 2013). The GABA switch relies on a developmentally regulated expression of the Na^+-K^+-$2Cl^-$ cotransporter 1 (NKCC1) and the K^+-Cl^- cotransporter 2 (KCC2). Due to a high level of NKCC1, a Cl^- importer, immature neurons accumulate this anion and, upon $GABA_A$Rs opening, Cl^- efflux generates membrane depolarization. Conversely, mature neurons express higher levels of the Cl^- exporter KCC2 and the resulting lower intracellular Cl^- concentration drives Cl^- influx through $GABA_A$Rs and leads to hyperpolarization (Rivera et al., 1999). While the molecular players of the GABA switch are well characterized, the signals that trigger this event have been only partially clarified.

Oxytocin (Oxt), a hypothalamic neurohormone known for decades for promoting parturition and lactation and for its role in social behavior (Meyer-Lindenberg et al., 2011), has also been implicated in the GABA switch. Maternal Oxt was reported to regulate the first transient phase of the GABA switch in the newborns (Tyzio et al., 2006). Pre-delivery treatments with Oxt or with a selective NKCC1 inhibitor in a model of autism, the valproate rat, and in a model of fragile X, the *Fmr1*$^{-/-}$ mouse, were shown to rescue the altered GABA balance in pups and social behavioral deficits in adults (Tyzio et al., 2014). However, it is at present unknown if Oxt also plays a role on the postnatal phase of the GABA switch. To address this issue, we took advantage of the *Oxtr*$^{-/-}$ mouse, a genetic model devoid of the oxytocin receptor (Oxtr), the main molecular target of Oxt in the brain. We have previously shown that *Oxtr*$^{-/-}$ animals display an autistic-like phenotype, which includes social and cognitive deficits and increased susceptibility to seizures, compatible with an altered E/I balance (Sala et al., 2011). Our present findings indicate that Oxtr is indeed essential for the proper developmental increase of KCC2 and for the consequent switch in GABA activity. In particular, we found that Oxt directly modulates the insertion of KCC2 at the plasma membrane in an early and very narrow developmental time

This is an open access article under the CC BY license (http://creativecommons.org/licenses/by/4.0/).

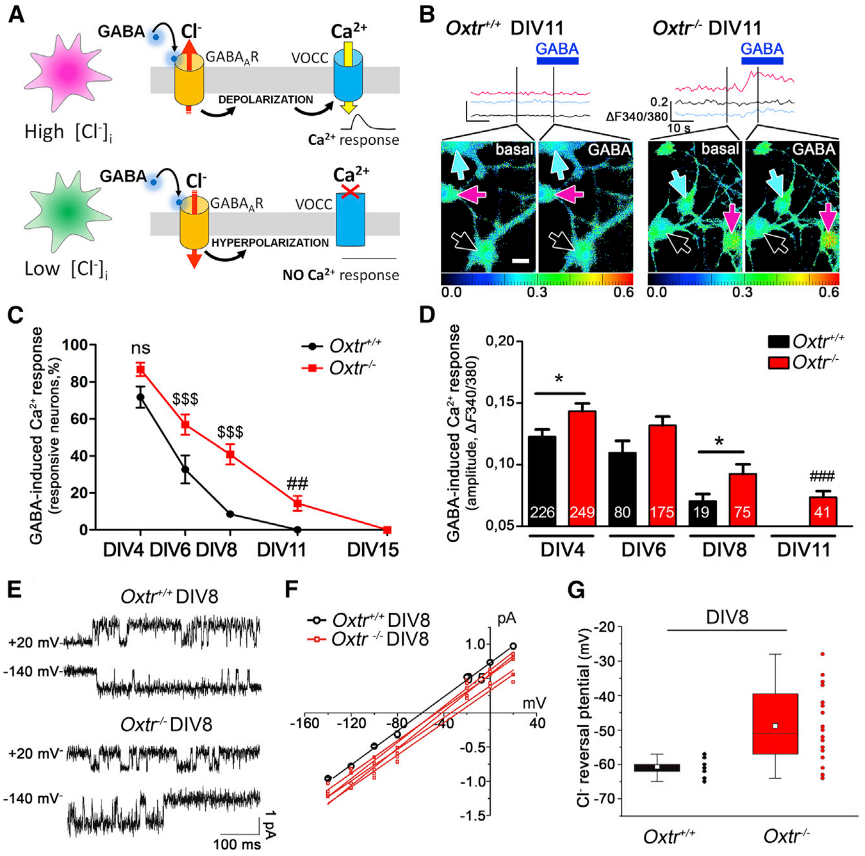

Figure 1. $Oxtr^{-/-}$ Neurons Have a Delayed GABA Switch

(A) Schematic representation of depolarizing and hyperpolarizing $GABA_AR$-induced responses monitored by Ca^{2+} imaging. In neurons with high $[Cl^-]_i$ (pink), GABA-induced depolarization opens VOCCs, leading to a Ca^{2+} peak. In neurons with low $[Cl^-]_i$ (green), GABA determines hyperpolarization, VOCCs remain closed, and no Ca^{2+} response is induced.

(B) Representative traces of $[Ca^{2+}]_i$ variations in the soma of DIV11 neurons in basal conditions and upon 100 μM GABA administration. Each trace refers to the recorded cell pointed by the color-matched arrow; pseudocolor scale from blue to red indicates increasing $[Ca^{2+}]$. Scale bar represents 10 μm.

(C) Percentage of $Oxtr^{+/+}$ and $Oxtr^{-/-}$ neurons showing GABA-induced Ca^{2+} responses along development. Data are from at least two different preparations (five to ten coverslips).

(D) Amplitude of GABA-induced Ca^{2+} peaks in $Oxtr^{+/+}$ and $Oxtr^{-/-}$ neurons at different stages of development. Number of responsive cells is reported in the bars. See Figure S1A for KCl-induced responses.

(E–G) Cell-attached recordings of single $GABA_A$ receptor in $Oxtr^{+/+}$ and $Oxtr^{-/-}$ hippocampal neuron at DIV8.

(E) Single channel current traces at +20 and −140 mV membrane potential in $Oxtr^{+/+}$ (top) and $Oxtr^{-/-}$ (bottom) neurons.

(F) i/V relationship of average $Oxtr^{+/+}$ single-channel recordings (n = 8; conductance 12 ± 0.2 pS) and five single experiments from $Oxtr^{-/-}$ neurons (average conductance 12 ± 0.6). Current reversal potential, obtained by linear fitting of the experimental data, was adjusted in each cell according to the measured resting membrane potential.

(G) Chart plot of single-channel current reversal potential for $Oxtr^{+/+}$ (n = 14) and $Oxtr^{-/-}$ (n = 22) neurons. See Figure S1B for resting membrane potentials at DIV8.

Data are presented as mean ± SEM; two-way ANOVA (Bonferroni post hoc test, $^{\$\$}p < 0.01$ and $^{\$\$\$}p < 0.001$; one-sample t test, $^{\#\#}p < 0.01$ and $^{\#\#\#}p < 0.001$; Student's t test, $^{*}p < 0.05$; and Student's t test with Welch's correction, $^{\&\&\&}p < 0.0001$). All mean, SEM, n, and p values are listed in Table S1.

window, thus affecting the GABA switch and neuronal excitability in a critical period for neuronal maturation.

RESULTS

Delayed GABA Switch in $Oxtr^{-/-}$ Hippocampal Neurons

The timing of the GABA switch can be monitored, in developing neuronal cultures, by measuring the occurrence and amplitude of GABA-induced Ca^{2+} responses (Figures 1A and 1B). To disclose any temporal difference in the occurrence of the GABA switch between $Oxtr^{-/-}$ and $Oxtr^{+/+}$ cultures, we evaluated, during development, the percentage of neurons showing GABA-induced Ca^{2+} transients and the amplitude of such responses (Figures 1C and 1D). At all time points, we found a significantly larger proportion of $Oxtr^{-/-}$ neurons increasing Ca^{2+} upon GABA stimulation (two-way ANOVA: genotype effect, $F(1,67) = 28.95$, $p < 0.0001$; time effect, $F(4,67) = 86.51$, $p < 0.0001$). Most interestingly, in $Oxtr^{+/+}$ neurons, the GABA-induced Ca^{2+} transients were completely lost at days in vitro 11 (DIV11), whereas in $Oxtr^{-/-}$ neurons, they disappeared only at DIV15 (Figure 1C).

Moreover, the amplitude of the Ca^{2+} responses was significantly higher in $Oxtr^{-/-}$ than in $Oxtr^{+/+}$ neurons (Figure 1D; two-way ANOVA: genotype effect, $F(1,827) = 5.56$, $p = 0.0186$; time effect, $F(2,827) = 11.41$, $p < 0.0001$), suggesting a stronger Cl^- gradient. A greater amplitude of Ca^{2+} responses could be caused also by an increased VOCC expression. However, this possibility can be excluded in $Oxtr^{-/-}$ neurons, since Ca^{2+} transients evoked by the administration of KCl (50 mM) were not augmented and were significantly reduced at DIV8 and DIV11 (Figure S1A).

Consistent with calcium measurements, cell-attached recordings of single $GABA_A$ receptor at DIV8 showed a difference in the reversal potential between $Oxtr^{+/+}$ and $Oxtr^{-/-}$ (Figures 1E and 1F). The range of $GABA_A$ reversal potential was significantly different in $Oxtr^{-/-}$ neurons (between −30 and −60 mV) than in $Oxtr^{+/+}$ (between −57 and −65 mV; t test with Welch's correction, $p < 0.0001$) (Figure 1G), even in the presence of a not significantly different resting membrane potential in the two populations (Figure S1B).

Altogether, these data indicate that in $Oxtr^{-/-}$ neurons, the GABA switch is delayed.

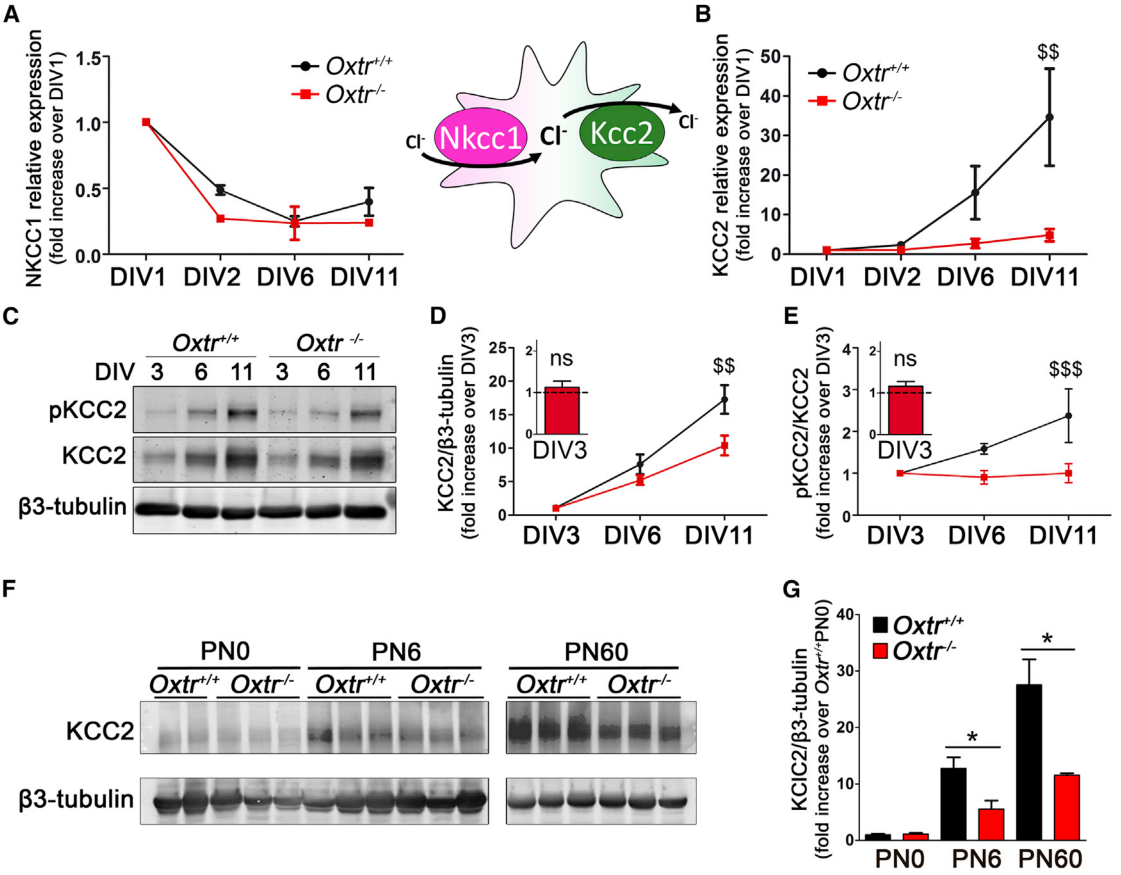

Figure 2. ***Oxtr*$^{-/-}$ Neurons Have Impaired KCC2 Expression**

(A and B) NKCC1 (A) and KCC2 (B) transcript levels during in vitro development measured by real-time qPCR analysis in three or four independent preparations and normalized on DIV1. The cartoon depicts the directions of Cl^- transport by NKCC1 and KCC2.

(C) Representative immunoblot of neuronal lysates at DIV3, DIV6, and DIV11 probed for p-Ser940KCC2 (pKCC2) and total KCC2.

(D and E) Quantification of total KCC2 normalized on β3-tubulin (D; n = 6) and of pKCC2 normalized on total KCC2 (E; n = 9) shown as fold increase over DIV3. Insets show no difference at DIV3 between *Oxtr*$^{-/-}$ (red bars) and *Oxtr*$^{+/+}$ (dotted lines).

(F and G) Immunoblot (F) and relative quantification (G) of hippocampal KCC2 expression at PN0, PN6, and PN60.

Data are presented as mean ± SEM; two-way ANOVA (Bonferroni post hoc test, $^{\$\$}p < 0.01$ and $^{\$\$\$}p < 0.001$; one-sample t test, $^{\#\#}p < 0.01$ and $^{\#\#\#}p < 0.001$; and Student's t test, $^{*}p < 0.05$). All mean, SEM, n, and p values are listed in Table S2.

Impaired KCC2 Upregulation and Phosphorylation in *Oxtr*$^{-/-}$ Neurons

In developing neurons, downregulation of NKCC1 and upregulation of KCC2 both contribute to reduce $[Cl^-]_i$; as a consequence, altered KCC2 and/or NKCC1 expression can be responsible for the delayed GABA switch in *Oxtr*$^{-/-}$ neurons. We thus investigated by real-time qPCR the expression profile of these transporters in *Oxtr*$^{-/-}$ and *Oxtr*$^{+/+}$ neurons from DIV1 to DIV11 (Figures 2A and 2B). The NKCC1 transcript was similarly downregulated in the two neuronal cultures (Figure 2A; two-way ANOVA: genotype effect, ($F(1,14) = 4.13$, $p > 0.05$). On the contrary, the KCC2 transcript underwent strong upregulation (30-fold) in *Oxtr*$^{+/+}$ neurons, while it barely increased by 5-fold in *Oxtr*$^{-/-}$ neurons (Figure 2B; two-way ANOVA: genotype effect, ($F(1,23) = 8.91$, $p = 0.0066$). Moreover, reduced KCC2 protein expression was observed in cultured *Oxtr*$^{-/-}$ neurons (Figures 2C and 2D; two-way ANOVA: genotype effect, $F(1,29) = 9.37$, $p < 0.0047$). These findings point to a defect in KCC2 upregulation as a main factor for the delayed GABA switch in *Oxtr*$^{-/-}$ neurons. Consistently, a KCC2 deficit was also found in hippocampal tissues from postnatal day 6 (PN6) and PN60 *Oxtr*$^{-/-}$ mice (Figures 2F and 2G).

We then analyzed KCC2 phosphorylation at Ser940 (pKCC2), a post-translational modification that stabilizes KCC2 at the cell surface and correlates with its cellular activity (Lee et al., 2007). We found a significant reduction of pKCC2 in *Oxtr*$^{-/-}$ versus *Oxtr*$^{+/+}$ neurons (Figures 2C and 2E; two-way ANOVA: genotype effect, $F(1,45) = 13.63$, $p = 0.0006$). The ratio between pKCC2 and KCC2 (Figure 2E) is an index of the amount of cotransporter at the plasma membrane. Between DIV3 and DIV11, this ratio underwent a 2-fold increase in *Oxtr*$^{+/+}$ neurons, whereas it remained constant in *Oxtr*$^{-/-}$ cells. These data suggest that Oxtr deficits affect both KCC2 expression and phosphorylation.

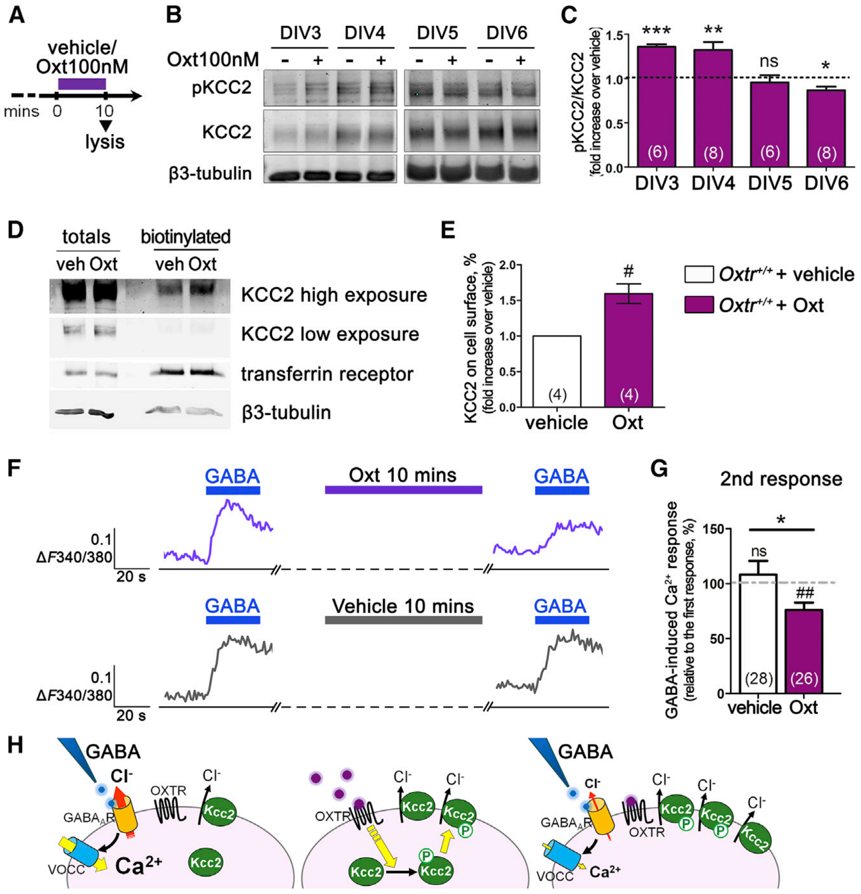

Figure 3. Oxt Increases KCC2 Phosphorylation, Promotes Its Insertion in the Plasma Membrane, and Reduces Excitatory GABA Responses at DIV3 and DIV4, but Not Later

(A) Scheme of Oxt administration: 100 nM Oxt (or vehicle) was administered to neurons 10 min before lysis.

(B) Representative immunoblots of pKCC2 and total KCC2. β3-tubulin was used as loading control.

(C) Quantification of pKCC2/total KCC2 as fold increase over vehicle treated age-matched samples (dotted line). See Figure S2A for *Oxtr* expression during development and Figures S2B–S2E for pKCC2 levels in $Oxtr^{+/+}$ and $Oxtr^{-/-}$ neurons upon Oxt administration.

(D) Immunoblot of a representative biotinylation experiment on DIV4 $Oxtr^{+/+}$ neurons. β3-tubulin and transferrin receptor were used as loading controls for total lysates (15 μg) and biotinylated surface proteins (150 μg).

(E) Quantification of biotinylated samples obtained by normalization on respective total lysates and correction for the amounts loaded. Data shown as fold increase over vehicle-treated samples.

(F) Representative temporal plots of GABA-induced $[Ca^{2+}]_i$ changes in an Oxt-treated (purple trace) and a vehicle-(gray trace) neuron.

(G) Percent variation in the amplitude (Δ*F*340/380) of GABA-induced responses upon Oxt or vehicle administration, calculated in individual cells. See Figure S2F for KCl-induced responses at the end of Calcium-imaging experiments.

(H) Modulation by Oxt of the GABA-induced Ca^{2+} response in $Oxtr^{+/+}$ neurons at DIV4. From the left: GABA administration induces a Ca^{2+} response via $GABA_AR$ and VOCC opening; 10-min Oxt treatment increases KCC2 phosphorylation, promotes its membrane insertion/stabilization, and increases Cl^- extrusion; a second GABA administration elicits a smaller depolarization and a reduced Ca^{2+} response. Data are presented as mean ± SEM; n numbers in brackets. Student's t test: $^*p < 0.05$, $^{**}p < 0.01$, $^{***}p < 0.001$; one-sample t test: $^{\#}p < 0.05$ and $^{\#\#}p < 0.01$. All mean, SEM, n, and p values are listed in Table S3.

Oxt Promoted Phosphorylation, Plasma Membrane Expression, and Function of KCC2 in a Restricted Developmental Time Window

To test for direct effects of Oxt on KCC2 insertion/stabilization at the plasma membrane, we evaluated the pKCC2/KCC2 ratio in $Oxtr^{+/+}$ neurons treated with 100 nM Oxt for 10 min (Figure 3A). Oxt significantly increased the pKCC2/KCC2 ratio at DIV3 and DIV4; however, at DIV5, Oxt failed to induce any increase in pKCC2, and a small reduction was observed at DIV6 (Figures 3B and 3C). Oxt-induced action was dose dependent, being Oxt-active down to a concentration of 1 nM (Figures S2B and S2C), and required the presence of Oxtr (Figures S2D and S2E). These findings indicate that in cultured neurons, Oxt, through its receptor, promotes KCC2 phosphorylation only in a very early and restricted time window.

Oxt-promoted membrane insertion of KCC2 was then verified by surface biotinylation experiments on DIV4 neurons. As shown in Figures 3D and 3E, 10-min treatment with 100 nM Oxt increased surface KCC2 by almost 60%.

Finally, to test if an increased membrane KCC2 could alter neuronal responses to GABA, we measured GABA-induced Ca^{2+} transients in $Oxtr^{+/+}$ neurons before and after Oxt application. Ca^{2+} rises were significantly blunted after Oxt administration (purple trace in Figures 3F and 3G) but not after vehicle treatment (gray trace in Figures 3F, 3G, and S2F). These findings indicate that Oxt is able to reduce GABA-induced depolarization by promoting KCC2 insertion/stabilization at the neuronal plasma membrane (Figure 3H).

Oxt Phosphorylation of KCC2 at Ser940 Was Mediated by an Oxtr/Gq/PKC-Dependent Pathway

We characterized the signaling pathways involved in the Oxt-dependent KCC2 phosphorylation at Ser940. Pretreatment of DIV4 $Oxtr^{+/+}$ neurons with the selective Gq-inhibitor YM254890 fully abolished the Oxt-mediated increase in pKCC2, indicating that Gq activation is required for the Oxt-induced effect. Moreover, the administration of YM254890 per se induced a decrease in pKCC2, suggesting that constitutive Gq activation is involved in the modulation of KCC2 phosphorylation at Ser940 (Figure 4A).

Administration of 100 nM Oxt to DIV4 $Oxtr^{+/+}$ neurons determined a robust phosphorylation of extracellular signal-regulated kinase (ERK), a known downstream target of Oxtr (Rimoldi et al., 2003) (Figures S3A and S3B). Neither basal nor Oxt-induced

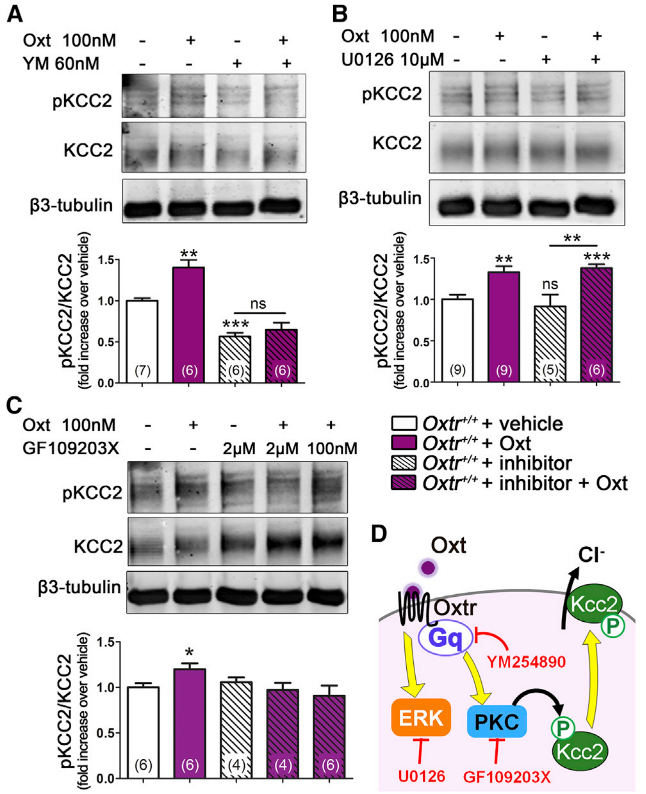

Figure 4. Oxytocin Increases KCC2 Phosphorylation on Ser940 via a Gq- and PKC-Dependent Pathway

(A–C) Representative immunoblots and relative quantifications of pKCC2 in neurons treated with vehicle (white bars) or 100 nM Oxt (purple bars) for 10 min, in the absence (plain bars) or presence (striped bars) of selective inhibitors: (A) Gq inhibitor YM254890 (YM, 60 nM, 5-min pretreatment), (B) MEK inhibitor U0126 (10 μM, 30-min pretreatment), and (C) PKC inhibitor GF109203X (GF, 2 μM or 100 nM, 20-min pretreatment). pKCC2 bands intensities were normalized over total KCC2 and displayed as fold change over vehicle. β3-tubulin was used as loading control. See Figure S3 for Oxt-induced ERK phosphorylation and for PMA-induced KCC2 phosphorylation.

(D) The cartoon shows the cellular pathway linking the Oxtr to the insertion/stabilization of KCC2 in the plasma membrane and the targets of the inhibitors used.

Data are presented as mean ± SEM; n numbers in brackets. Student's t test: *p < 0.05, **p < 0.01, and ***p < 0.001. All mean, SEM, n, and p values are listed in Table S4.

pKCC2 levels were modified by U0126, an inhibitor of ERK phosphorylation (Figure 4B), indicating that ERK activation is not required for KCC2 phosphorylation at Ser940.

Protein kinase C (PKC), a downstream effector of Gq, has been reported to directly phosphorylate Ser940 and to increase the surface expression of KCC2 (Bos et al., 2013; Kahle et al., 2013; Lee et al., 2007). We confirmed that PKC induces KCC2 phosphorylation at early (DIV4 abd DIV6) and late (DIV11) stages of in vitro development by applying phorbol 12-myristate 13-acetate (PMA), a PKC activator, to *Oxtr*$^{+/+}$ neurons (Figures S3C and S3D). To determine if Oxt administration at DIV4 affects KCC2 phosphorylation levels through PKC activation, pKCC2 levels were determined after a pretreatment with the PKC inhibitor GF109203X at 100 nM (to selectively block only the conventional α and β PKC isoforms), and at 2 μM (to inhibit conventional and novel PKC isoforms; Martiny-Baron et al., 1993) (Figure 4C). Both concentrations of GF109203X prevented the Oxt-induced increase in pKCC2, demonstrating that the conventional isoforms of PKC are involved in the Oxtr/Gq-mediated pathway of KCC2 phosphorylation (Figure 4D).

Mature *Oxtr*$^{-/-}$ Hippocampal Neurons Displayed an Altered E/I Balance

Due to the relevance of the GABA switch on development and function of neuronal networks, the consequences of the delayed GABA switch observed in *Oxtr*$^{-/-}$ neurons may persist beyond immature stages. To address this issue, we analyzed the morphological and electrophysiological properties of mature *Oxtr*$^{-/-}$ neurons in culture. No differences were found in the number and morphology of dendritic spines (Figure S4) or in resting membrane potential between *Oxtr*$^{-/-}$ and *Oxtr*$^{+/+}$ neurons (Figure 5A). Normal functional responses to the chemically induced long-term potentiation (LTP) were observed, indicating normal plasticity of excitatory synapses (Figures 5B and 5C). We then looked for possible alterations of neuronal functions by recording miniature excitatory and inhibitory postsynaptic currents (mEPSCs and mIPSC, respectively; Figure 5D). Measurement of mEPSCs revealed a significant increase in the frequency of excitatory events in *Oxtr*$^{-/-}$ neurons that was not associated with changes in the mean amplitude and quantal charge (Figures 5E–5G). The same analysis performed on inhibitory events revealed no changes in terms of mean frequency but a significant decrease in the amplitude values of *Oxtr*$^{-/-}$ neurons (Figures 5H and 5I) accompanied by a significantly reduced quantal charge (Figure 5J). These results indicate an unbalance between excitation and inhibition in *Oxtr*$^{-/-}$ neurons, which show indeed a much higher E/I ratio, calculated for each individual cell by dividing the frequencies of mEPSCs by those of mIPSCs (Figure 5K).

DISCUSSION

Our data indicate that, in the absence of *Oxtr*, the developmental upregulation of KCC2 is impaired and the GABA switch is delayed. Alterations in the timing of the GABA switch have been previously reported in *Fmr1*$^{-/-}$ mice, a model of fragile X syndrome, and in rats exposed in utero to valproate (VPA), a model of autism (He et al., 2014; Tyzio et al., 2014). Here, we found an analogous impairment in neurons from the *Oxtr*$^{-/-}$ mouse, a model of autism itself (Sala et al., 2011), in line with the hypothesis that a delayed GABA switch may be a feature shared by several neurodevelopmental disorders. Electrophysiological and behavioral deficits in *Fmr1*$^{-/-}$ and VPA animals have been successfully restored by selective drugs targeting either the Oxt system or the Cl^- cotransporters (Eftekhari et al., 2014; Tyzio et al., 2014). However, the link between these two players of the GABA switch remained unsolved.

Here, we show for the first time that the lack of Oxtr in neurons affects specifically KCC2 without impairing NKCC1. Interestingly, both Oxt and KCC2 modulates the GABAergic system. Oxt promotes the rapid formation of inhibitory synapses in adult hypothalamic GABAergic neurons (Theodosis et al., 2006), while

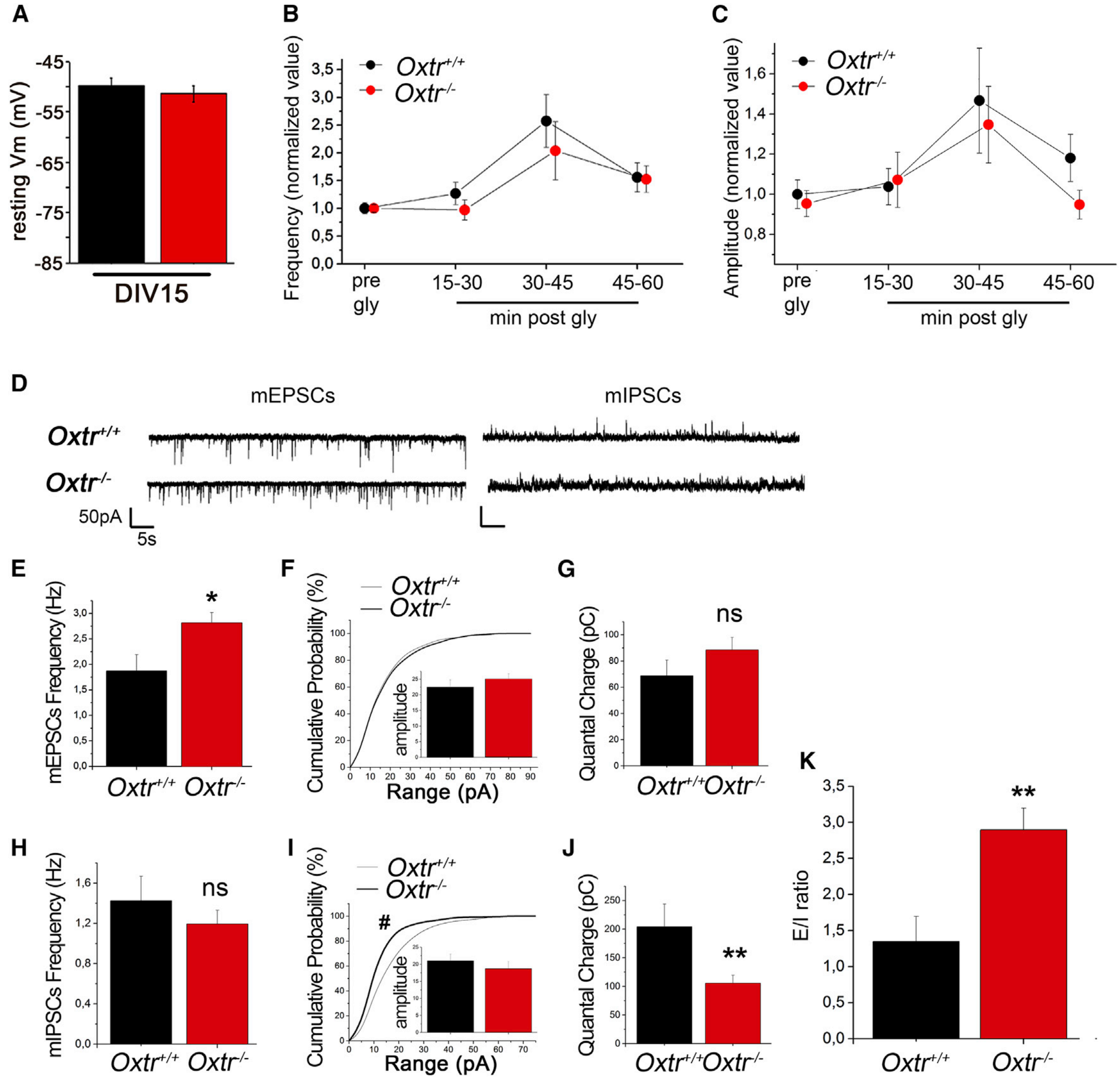

Figure 5. Mature *Oxtr*$^{-/-}$ Neurons Have Normal Resting Vm but Display an Impaired E/I Ratio

(A) Measurement of resting membrane potential (Vm) from DIV14 hippocampal neurons did not show any differences between *Oxtr*$^{+/+}$ and *Oxtr*$^{-/-}$ groups.

(B–K) Electrophysiological characterization of neurons at DIV14. (B) Frequency and (C) amplitude of chemically induced LTP. (D) Representative traces of spontaneous mEPSCs and mIPSCs. Quantification of (E) mEPSC and (H) mIPSCs frequencies. Analysis of (F) mEPSC and (I) mIPSC amplitudes shown as cumulative distribution and mean value (insert). Mean quantal charge transferred during individual (G) mEPSCs and (J) mIPSCs. (K) Excitatory/inhibitory ratio evaluated on the frequencies of miniature events in individual neurons, as described in Supplemental Experimental Procedures. See Figure S4 for dendritic spines analysis.

Data are presented as mean ± SEM. Student's t test: *p < 0.05 and **p < 0.01; and Kolmogorov-Smirnov test on cumulative distribution: $^{\#}$p < 0.05. All mean, SEM, n, and p values are listed in Table S5.

KCC2 is fundamental for the maturation of interneurons (Bortone and Polleux, 2009), and its overexpression can increase the density of inhibitory synapses and $GABA_AR$ clusters (Chudotvorova et al., 2005). We previously reported, in *Oxtr*$^{-/-}$ neurons, an increased ratio between excitatory and inhibitory synapses (Sala et al., 2011), originating from an increase in excitatory synapses and/or a decrease in the inhibitory ones. We report here an increase in the number of functional glutamatergic presynaptic boutons (as suggested by the increased mEPSC frequency) in the presence of unaltered GABA presynaptic input (as suggested by the unchanged mIPSC frequency). An alteration at inhibitory postsynapses in *Oxtr*$^{-/-}$ neurons was revealed by the decreased quantal charge and amplitude of mIPSCs, most likely arising from a change in Cl^- homeostasis and $GABA_A$ reversal potential. An increased excitatory presynaptic neurotransmission coupled with a reduced postsynaptic inhibition are thus at the basis of the E/I unbalance observed in *Oxtr*$^{-/-}$ neurons.

Our present data indicate that KCC2 is one of the molecular players through which the Oxt system could increase the inhibitory tone in hippocampal neurons. However, different effects of Oxt on neurotransmission during development have been

observed in different regions; for instance, in the sensory (but not in the prefrontal) cortex, Oxt was found to promote excitatory, rather than inhibitory, activity (Zheng et al., 2014).

Very importantly, a role of Oxt in postnatal brain development is emerging as a relevant novel aspect of this neuropeptide (Grinevich et al., 2014). Indeed, the Oxt system was found to be involved in experience-dependent development of sensory cortices, a process occurring in a restricted critical period of early postnatal life (Zheng et al., 2014).

Within this line of evidence, our finding that Oxt modulates KCC2 expression/stabilization at the plasma membrane only in a very early and narrow time window (by DIV4) is particularly intriguing. The subsequent disappearance of this Oxt effect apparently depends not on a downregulation of its receptor, as real-time qPCR in *Oxtr*$^{+/+}$ neurons reveals a developmental upregulation of the Oxtr transcript (up to 8-fold by DIV11; Figure S2A), but most likely on a change in the Oxtr-activated pathway. Analogous age-dependent effects on KCC2 were described for brain-derived neurotrophic factor, being it a positive modulator of KCC2 transcription in immature neurons and a repressor in mature ones (Choe et al., 2015; Ludwig et al., 2011; Rivera et al., 2002).

Oxt actions in early postnatal events seem therefore to result in unique outcomes, which may depend, among other factors, on the specific signaling pathways activated. Oxtr is a promiscuous receptor coupled to several G protein isoforms, triggering multiple signaling cascades (Busnelli et al., 2012). Here, we show that the Gq/PKC pathway in the first stages of development is involved in KCC2 phosphorylation at Ser940, a modification that confers KCC2 with a higher stability at the cell surface, increasing its activity and reducing GABA-induced depolarization. Oxtr-activated pathways may change in the following stages of development inducing different effects in more mature neurons. Elucidating the heterogeneity of Oxt-induced responses is compelling, since this peptide has been proposed as a treatment in a number of neurodevelopmental and neuropsychiatric conditions (Grinevich et al., 2014; Meyer-Lindenberg et al., 2011). Although exogenous Oxt administration to adult autistic patients inconsistently improved symptoms (Young and Barrett, 2015), it proved successful on the onset and progression of neurodevelopmental dysfunctions when applied at birth in animal models of autism and Prader-Willi syndrome (Peñagarikano et al., 2015; Tyzio et al., 2014; Meziane et al., 2015).

The identification of KCC2 as an Oxt target provides a better understanding of the role and therapeutic potential of Oxt for the treatment of neurodevelopmental disorders.

EXPERIMENTAL PROCEDURES

Animals and Primary Hippocampal Cultures

Oxtr$^{+/+}$ and *Oxtr*$^{-/-}$ mice (Takayanagi et al., 2005) were rederived on a C57BL/6 genetic background. All animal procedures were approved by the Italian Ministry of Health (authorization no. 295/2012A; December 20, 2012; protocol no. 1/2014). All experiments were performed in accordance with the Italian legislation. Embryonic day 18 dissociated hippocampal neurons were obtained as described in the Supplemental Experimental Procedures.

Calcium Imaging and Electrophysiology

For calcium imaging recordings, hippocampal neurons were loaded with Fura-2/AM. $[Ca^{2+}]_i$ was measured as the fluorescence *F*340/380 ratio. Changes over baseline (ΔF340/380) higher than 0.05 units in response to 100 μM GABA were considered depolarizing events.

$GABA_A$ reversal potential was calculated from single-channel recordings in cell-attached configuration in DIV8 neuron and corrected for the recorded membrane potential. Whole-cell patch-clamp recordings were performed in DIV14 neurons. mEPSCs and mIPSCs were recorded by holding neurons at the reversal potential for GABAergic (−70 mV) and glutamatergic (+10 mV) responses in a solution containing 1 μM TTX. See the Supplemental Experimental Procedures for details.

Biochemistry and Molecular Biology

For biotinylation assays, neurons were incubated with ice-cold EZ-Link-Sulfo-NHS-LC-biotin (1 mg/ml), quenched with 50 mM glycine, lysed, and loaded on Streptavidin beads. Biotinylated proteins were separated on SDS-polyacrylamide gels. In western blotting experiments, specific bands were visualized with secondary antibodies conjugated with infrared-emitting fluorophores and signals were quantified using an Odyssey scanner (Li-Cor). For real-time qPCR, cDNA samples were amplified using TaqMan Gene Expression Assay probes. See the Supplemental Experimental Procedures for details.

Statistics

Statistical analysis was performed with GraphPad Prism5 software. The tests used to assess data significance are indicated in the figure legends. For details, see the Supplemental Experimental Procedures.

SUPPLEMENTAL INFORMATION

Supplemental Information includes Supplemental Experimental Procedures, four figures, and five tables and can be found with this article online at http://dx.doi.org/10.1016/j.celrep.2016.03.013.

AUTHOR CONTRIBUTIONS

M.L. designed and conducted cellular, biochemical, and calcium imaging experiments. M.B. contributed to neuronal culture preparation. F.A. and M.M. designed and performed electrophysiological experiments. C.V. supervised the calcium imaging experiments. B.C. conceived and supervised the project. All authors wrote the manuscript and gave final approval for publication.

ACKNOWLEDGMENTS

This work was supported by the Telethon Foundation (grant GGP12207) and the CNR Research Project on Aging (to B.C.) and FIRB-RBFR10ZBYZ (to F.A.). M.L. was the recipient of a Fondazione Fratelli Confalonieri postdoctoral fellowship. We thank C. Rivera (INSERM) for the KCC2 antibody and V. Gigliucci for critically reading the manuscript.

Received: September 23, 2015
Revised: February 5, 2016
Accepted: February 27, 2016
Published: March 24, 2016

REFERENCES

Ben-Ari, Y., Cherubini, E., Corradetti, R., and Gaiarsa, J.L. (1989). Giant synaptic potentials in immature rat CA3 hippocampal neurones. J. Physiol. *416*, 303–325.

Bortone, D., and Polleux, F. (2009). KCC2 expression promotes the termination of cortical interneuron migration in a voltage-sensitive calcium-dependent manner. Neuron *62*, 53–71.

Bos, R., Sadlaoud, K., Boulenguez, P., Buttigieg, D., Liabeuf, S., Brocard, C., Haase, G., Bras, H., and Vinay, L. (2013). Activation of 5-HT2A receptors upregulates the function of the neuronal K-Cl cotransporter KCC2. Proc. Natl. Acad. Sci. USA *110*, 348–353.

Busnelli, M., Saulière, A., Manning, M., Bouvier, M., Galés, C., and Chini, B. (2012). Functional selective oxytocin-derived agonists discriminate between individual G protein family subtypes. J. Biol. Chem. *287*, 3617–3629.

Choe, K.Y., Han, S.Y., Gaub, P., Shell, B., Voisin, D.L., Knapp, B.A., Barker, P.A., Brown, C.H., Cunningham, J.T., and Bourque, C.W. (2015). High salt intake increases blood pressure via BDNF-mediated downregulation of KCC2 and impaired baroreflex inhibition of vasopressin neurons. Neuron *85*, 549–560.

Chudotvorova, I., Ivanov, A., Rama, S., Hübner, C.A., Pellegrino, C., Ben-Ari, Y., and Medina, I. (2005). Early expression of KCC2 in rat hippocampal cultures augments expression of functional GABA synapses. J. Physiol. *566*, 671–679.

Eftekhari, S., Shahrokhi, A., Tsintsadze, V., Nardou, R., Brouchoud, C., Conesa, M., Burnashev, N., Ferrari, D.C., and Ben-Ari, Y. (2014). Response to Comment on "Oxytocin-mediated GABA inhibition during delivery attenuates autism pathogenesis in rodent offspring". Science *346*, 176.

Grinevich, V., Desarménien, M.G., Chini, B., Tauber, M., and Muscatelli, F. (2014). Ontogenesis of oxytocin pathways in the mammalian brain: late maturation and psychosocial disorders. Front. Neuroanat. *8*, 164.

He, Q., Nomura, T., Xu, J., and Contractor, A. (2014). The developmental switch in GABA polarity is delayed in fragile X mice. J. Neurosci. *34*, 446–450.

Kahle, K.T., Deeb, T.Z., Puskarjov, M., Silayeva, L., Liang, B., Kaila, K., and Moss, S.J. (2013). Modulation of neuronal activity by phosphorylation of the K-Cl cotransporter KCC2. Trends Neurosci. *36*, 726–737.

Lee, H.H., Walker, J.A., Williams, J.R., Goodier, R.J., Payne, J.A., and Moss, S.J. (2007). Direct protein kinase C-dependent phosphorylation regulates the cell surface stability and activity of the potassium chloride cotransporter KCC2. J. Biol. Chem. *282*, 29777–29784.

Ludwig, A., Uvarov, P., Soni, S., Thomas-Crusells, J., Airaksinen, M.S., and Rivera, C. (2011). Early growth response 4 mediates BDNF induction of potassium chloride cotransporter 2 transcription. J. Neurosci. *31*, 644–649.

Martiny-Baron, G., Kazanietz, M.G., Mischak, H., Blumberg, P.M., Kochs, G., Hug, H., Marmé, D., and Schächtele, C. (1993). Selective inhibition of protein kinase C isozymes by the indolocarbazole Gö 6976. J. Biol. Chem. *268*, 9194–9197.

Meyer-Lindenberg, A., Domes, G., Kirsch, P., and Heinrichs, M. (2011). Oxytocin and vasopressin in the human brain: social neuropeptides for translational medicine. Nat. Rev. Neurosci. *12*, 524–538.

Meziane, H., Schaller, F., Bauer, S., Villard, C., Matarazzo, V., Riet, F., Guillon, G., Lafitte, D., Desarmenien, M.G., Tauber, M., and Muscatelli, F. (2015). An early postnatal oxytocin treatment prevents social and learning deficits in adult mice deficient for Magel2, a gene involved in Prader-Willi syndrome and autism. Biol. Psychiatry *78*, 85–94.

Peñagarikano, O., Lázaro, M.T., Lu, X.H., Gordon, A., Dong, H., Lam, H.A., Peles, E., Maidment, N.T., Murphy, N.P., Yang, X.W., et al. (2015). Exogenous and evoked oxytocin restores social behavior in the Cntnap2 mouse model of autism. Sci. Transl. Med. *7*, 271ra8.

Rimoldi, V., Reversi, A., Taverna, E., Rosa, P., Francolini, M., Cassoni, P., Parenti, M., and Chini, B. (2003). Oxytocin receptor elicits different EGFR/MAPK activation patterns depending on its localization in caveolin-1 enriched domains. Oncogene *22*, 6054–6060.

Rivera, C., Voipio, J., Payne, J.A., Ruusuvuori, E., Lahtinen, H., Lamsa, K., Pirvola, U., Saarma, M., and Kaila, K. (1999). The K+/Cl- co-transporter KCC2 renders GABA hyperpolarizing during neuronal maturation. Nature *397*, 251–255.

Rivera, C., Li, H., Thomas-Crusells, J., Lahtinen, H., Viitanen, T., Nanobashvili, A., Kokaia, Z., Airaksinen, M.S., Voipio, J., Kaila, K., and Saarma, M. (2002). BDNF-induced TrkB activation down-regulates the K+-Cl- cotransporter KCC2 and impairs neuronal Cl- extrusion. J. Cell Biol. *159*, 747–752.

Sala, M., Braida, D., Lentini, D., Busnelli, M., Bulgheroni, E., Capurro, V., Finardi, A., Donzelli, A., Pattini, L., Rubino, T., et al. (2011). Pharmacologic rescue of impaired cognitive flexibility, social deficits, increased aggression, and seizure susceptibility in oxytocin receptor null mice: a neurobehavioral model of autism. Biol. Psychiatry *69*, 875–882.

Takayanagi, Y., Yoshida, M., Bielsky, I.F., Ross, H.E., Kawamata, M., Onaka, T., Yanagisawa, T., Kimura, T., Matzuk, M.M., Young, L.J., and Nishimori, K. (2005). Pervasive social deficits, but normal parturition, in oxytocin receptor-deficient mice. Proc. Natl. Acad. Sci. USA *102*, 16096–16101.

Theodosis, D.T., Koksma, J.J., Trailin, A., Langle, S.L., Piet, R., Lodder, J.C., Timmerman, J., Mansvelder, H., Poulain, D.A., Oliet, S.H., and Brussaard, A.B. (2006). Oxytocin and estrogen promote rapid formation of functional GABA synapses in the adult supraoptic nucleus. Mol. Cell. Neurosci. *31*, 785–794.

Tyzio, R., Cossart, R., Khalilov, I., Minlebaev, M., Hübner, C.A., Represa, A., Ben-Ari, Y., and Khazipov, R. (2006). Maternal oxytocin triggers a transient inhibitory switch in GABA signaling in the fetal brain during delivery. Science *314*, 1788–1792.

Tyzio, R., Nardou, R., Ferrari, D.C., Tsintsadze, T., Shahrokhi, A., Eftekhari, S., Khalilov, I., Tsintsadze, V., Brouchoud, C., Chazal, G., et al. (2014). Oxytocin-mediated GABA inhibition during delivery attenuates autism pathogenesis in rodent offspring. Science *343*, 675–679.

Valeeva, G., Valiullina, F., and Khazipov, R. (2013). Excitatory actions of GABA in the intact neonatal rodent hippocampus in vitro. Front. Cell. Neurosci. *7*, 20.

Young, L.J., and Barrett, C.E. (2015). Neuroscience. Can oxytocin treat autism? Science *347*, 825–826.

Zheng, J.J., Li, S.J., Zhang, X.D., Miao, W.Y., Zhang, D., Yao, H., and Yu, X. (2014). Oxytocin mediates early experience-dependent cross-modal plasticity in the sensory cortices. Nat. Neurosci. *17*, 391–399.

Cell Reports, Volume *15*

Supplemental Information

The Timing of the Excitatory-to-Inhibitory GABA Switch Is Regulated by the Oxytocin Receptor via KCC2

Marianna Leonzino, Marta Busnelli, Flavia Antonucci, Claudia Verderio, Michele Mazzanti, and Bice Chini

SUPPLEMENTAL DATA

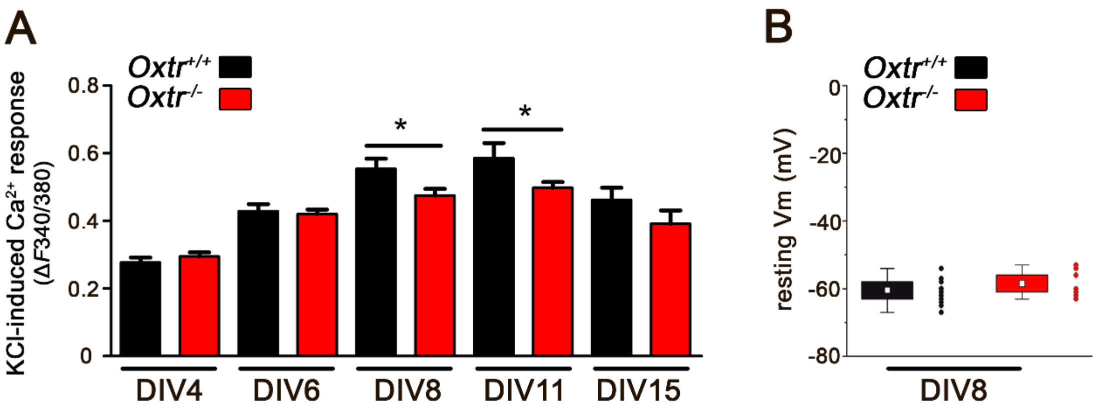

Figure S1 (related to Figure 1). Evaluation of KCl-induced depolarization and resting membrane potential in ***Oxtr*** $^{+/+}$ **and** ***Oxtr*** $^{-/-}$ **hippocampal neurons.**

(A) The amplitude of high K^{+}-induced Ca^{2+} responses, indicative of VOCC expression, was measured with Ca^{2+} imaging at DIV4, 6, 8, 11 and 15 and expressed as ΔF340/380. More than 30 neurons from at least 2 different preparations were analyzed per timepoint.

(B) Average resting membrane potential (Vm) of *Oxtr* $^{+/+}$ (n= 15) and *Oxtr* $^{-/-}$ (n= 9) single neurons.

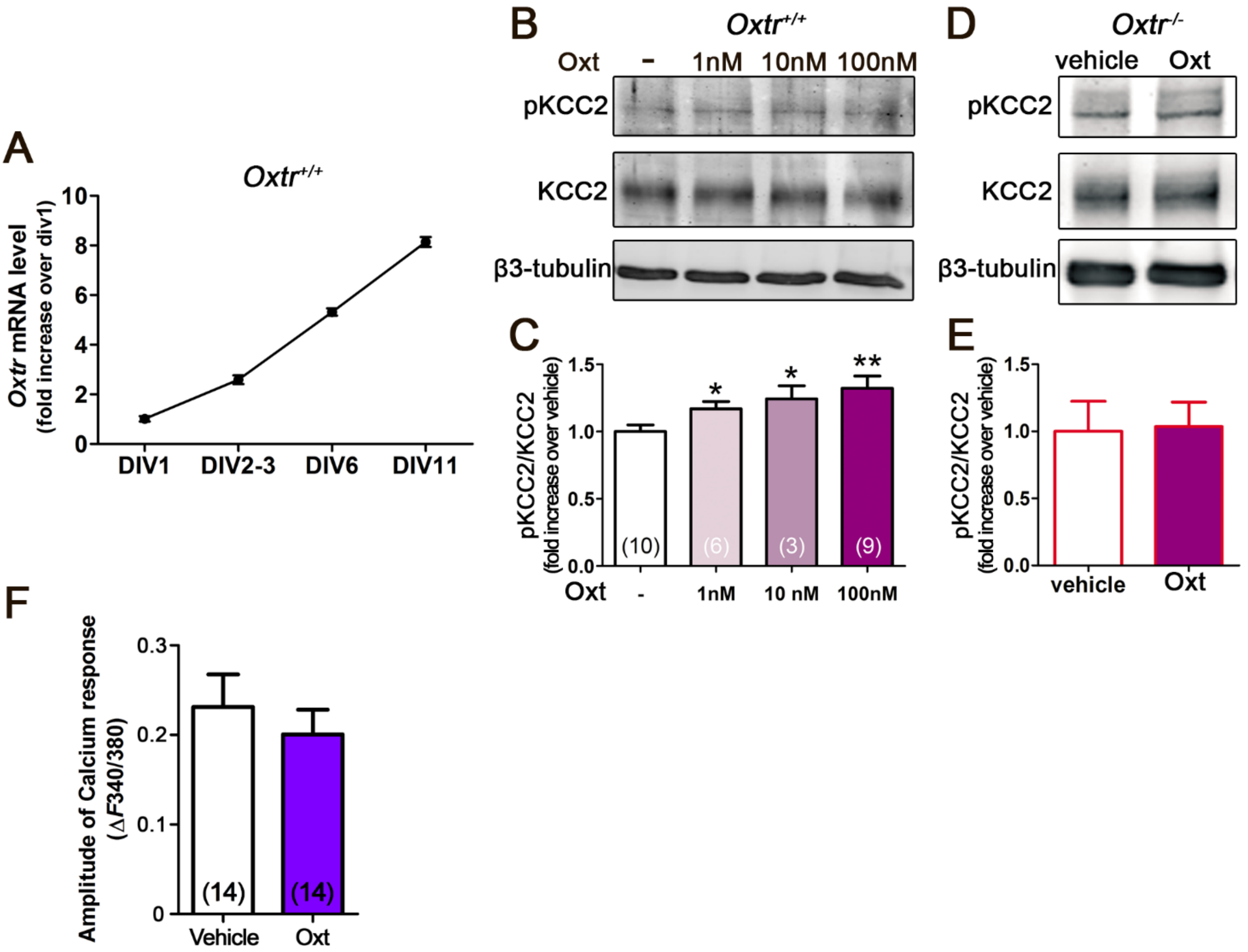

Figure S2 (related to Figure 3). Oxt modulates pKCC2 levels through the Oxytocin receptor in a dose-dependent way.

(A) Oxtr expression was detected in *Oxtr*$^{+/+}$ neurons already at DIV1 and increased over time. Relative levels of the transcript determined by qRT-PCR are shown as fold change over DIV1 (N=3). **(B-C)** Higher Oxt dose administered to DIV4 *Oxtr*$^{-+/+}$ neurons (10 min treatment) increased relative KCC2 phosphorylation. **(B)** Representative immunoblot and **(C)** quantification of pKCC2/total KCC2 shown as fold increase over vehicle treated age-matched samples. β3-tubulin was used as loading control. N numbers in brackets. **(D-E)** 10 min Oxt treatment (100nM) at DIV4 has no effects on *Oxtr*$^{-/-}$ neurons. **(D)** Representative immunoblot and **(E)** relative quantification of pKCC2/total KCC2 shown as fold increase over vehicle treated age-matched samples. β3-tubulin was used as loading control. (N=3). **(F)** Vehicle- and Oxt-treated neurons showed similar KCl-induced responses at the end of Calcium-imaging experiments, indicating that they both maintained a good neuronal viability. N numbers in brackets. Data are presented as mean ± SEM; Student *t* test: *p<0.05; **p<0.01.

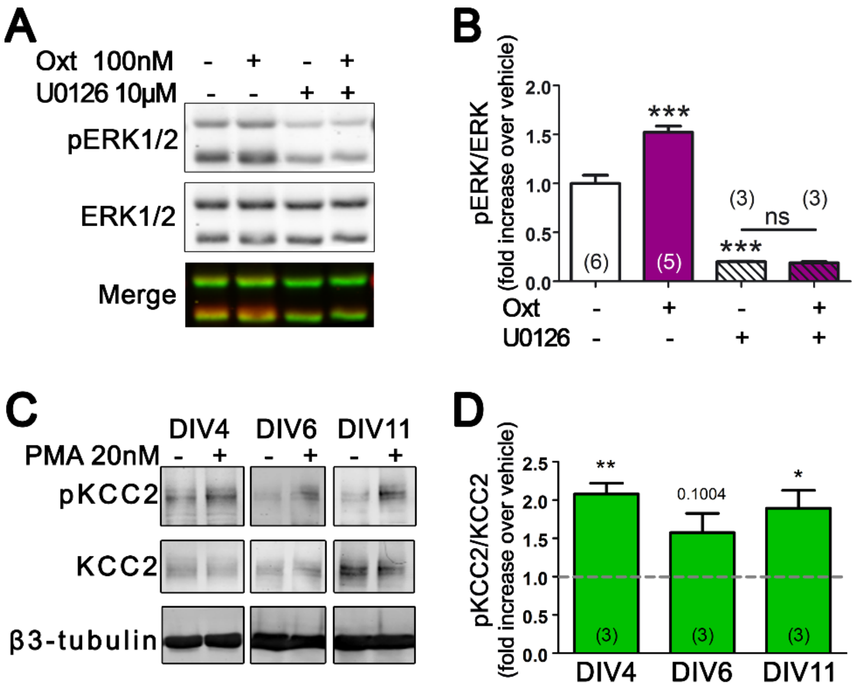

Figure S3 (related to Figure 4). Oxt-mediated signaling during development.
(A-B) Oxt treatment (100nM, 10 min) induces ERK1/2 phosphorylation in *Oxtr*$^{+/+}$ neurons at DIV4. **(A)** Immunoblot of phosphorylated and total ERK1/2 in *Oxtr*$^{+/+}$ neurons at DIV4 upon 10 min treatment with 100nM OXT (purple bars) in the absence (plain bars) or presence (patterned bars) of the MEK inhibitor U0126 (10µM, 30 min pretreatment). The signals coming from both IR-channels are shown in the "Merge" panel (red, p-ERK1/2-GaM680; green, total ERK1/2-GaR800). **(B)** Quantification of ERK1/2 phosphorylation calculated as the ratio between p-ERK1/2 and total ERK1/2 and displayed as fold change over vehicle-treated samples. **(C-D)** Exogenous activation of PKC with PMA (20nM, 30 min) increased KCC2 phosphorylation at DIV4 (*t*-test, p=0.056), DIV6 (even though not statistically significant; *t*-test, p=0.1004) and DIV11 (*t*-test, p=0.0219) indicating that KCC2 remains a target of PKC-mediated phosphorylation during in vitro development. **(C)** Immunoblot of phosphorylated and total KCC2 in *Oxtr*$^{+/+}$ neurons at DIV4, DIV6 and DIV11 treated with vehicle or PMA. **(D)** Quantification of KCC2 phosphorylation calculated as the ratio between p-KCC2 and total KCC2 and displayed as fold change over age-matched vehicle-treated samples (dotted line). Data presented as mean ± SEM; Student *t* test: *p<0.05; **p<0.01; ***p<0.001; N numbers in brackets.

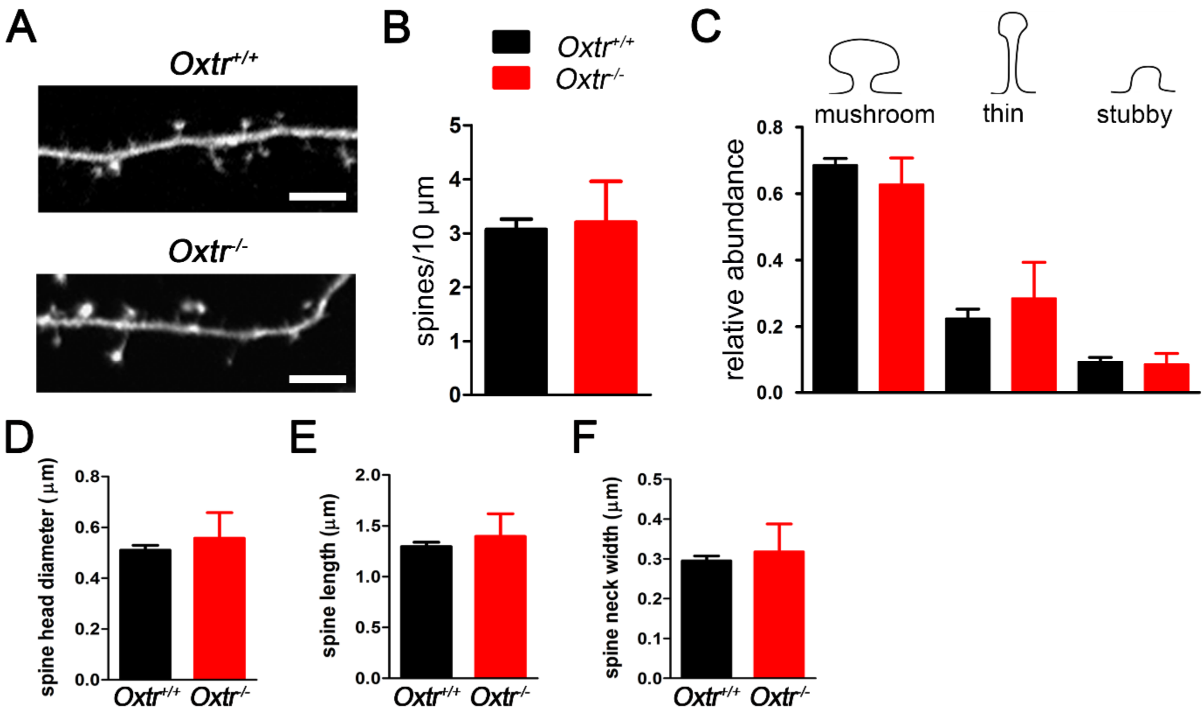

Figure S4 (related to Figure 5): Mature hippocampal neurons from *Oxtr*$^{+/+}$ and *Oxtr*$^{-/-}$ mice have similar spine density and morphology.

(A) Analysis of spine density and morphology at DIV17 in GFP-transfected neurons reveals no difference between *Oxtr*$^{+/+}$ and *Oxtr*$^{-/-}$ cells. Scale bars 10 µm. **(B)** The spine density was calculated as number of spine per 10µm of dendrite. **(C)** The abundance of different types of spines (mushroom, thin and stubby) was analyzed by classifying spines in categories based on morphological parameters: **(D)** spine head diameter, **(E)** spine length and **(F)** spine neck width. Data presented as mean ± SEM. Student *t* test: $p>0.05$. 5-6 neurons analyzed for each genotype.

Table S1. Mean values, SEM, number of samples and p values of data displayed in Figure 1

**Calcium imaging**	*Oxtr*$^{+/+}$			*Oxtr*$^{-/-}$			p value Bonferroni *post hoc* * Student *t*-test # One-sample *t*-test & Student *t*-test with Welch's correction
	mean	SEM	N	mean	SEM	N	
Figure 1C – GABA-responsive cells							
DIV4	71.7%	5.7	10	86.7%	3.6	8	>0.05
DIV6	32.6%	7.5	9	59.8%	5.2	8	<0.001
DIV8	8.5%	1.8	9	37.7%	5.3	6	<0.001
DIV11	0		5	14.3%	4.0	10	# 0.0051
DIV15	0		5	0		6	-
Figure 1D - ΔGABA							
DIV4	122.7	5.921	226	143.4	6.354	249	* 0.0182
DIV6	109.6	9.732	80	131.8	7.255	175	* 0.0691
DIV8	70.4	5.883	19	92.59	7.819	75	* 0.0261
DIV11				73.62	5.079	41	# <0.0001
**Electrophysiology – GABA reversal potential**							
Figure 1G	-60.71	0.665	14	-49.5	2.232	22	& <0.0001

Table S2. Mean values, SEM, number of samples and p values of data displayed in Figure 2

	Oxtr$^{+/+}$			*Oxtr*$^{-/-}$			p value<br>Bonferroni *post hoc*<br>* Student *t*-test<br># One-sample *t*-test
	mean	SEM	N	mean	SEM	N	
**Real Time**							
Figure 2A – NKCC1							
DIV2	0.4866	0.0352	3	0.2664	0.0193	2	>0.05
DIV6	0.25	0.0378	3	0.235	0.125	2	>0.05
DIV11	0.4066	0.1481	3	0.24	0.0208	3	>0.05
Figure 2B – KCC2							
DIV2	2.35	0.3871	4	1.09	0.3927	3	>0.05
DIV6	15.5625	6.6892	4	2.7375	1.1677	4	>0.05
DIV11	34.6325	12.2596	4	4.835	1.5706	4	<0.01
**Western Blot**							
Figure 2D							
DIV3 (inset)			12	1.16	0.1123	12	# 0.1824
DIV6	7.5859	1.4620	6	5.2268	0.7135	6	> 0.05
DIV11	17.2715	2.1280	6	10.4111	1.4670	5	<0.01
Figure 2E							
DIV3 (inset)			12	1.125	0.1501	12	# 0.4216
DIV6	1.5841	0.1283	9	0.9050	0.1585	9	>0.05
DIV11	2.3746	0.6405	6	1.0026	0.2271	9	<0.001
Figure 2G							
PN0	1.000	0.1880	2	1.132	0.1920	3	* 0.6753
PN6	12.76	1.963	3	5.575	1.488	3	* 0.0434
PN60	27.56	4.502	3	11.55	0.3355	3	* 0.0239

Table S3. Mean values, SEM, number of samples and p values of data displayed in Figure 3

	mean	SEM	N	p value<br>Student *t*-test<br># One-sample t-test
**WB- Oxt treatment -** Figure 3C				
DIV3	1.357	0.02986	6	<0.0001
DIV4	1.321	0.09216	8	0.0086
DIV5	0.9556	0.08156	6	0.7518
DIV6	0.8672	0.041	8	0.0418
**Biotinylation Assay -** Figure 3E				
Oxt	1.594	0.1379	4	# 0.0231
**Calcium imaging -** Figure 3G				
$Oxtr^{+/+}$	108.2	12.53	28	# 0.5173
Oxtr-/-	75.92	6.652	26	# 0.0013<br>0.0282

Table S4. Mean values, SEM, number of samples and p values of data displayed in Figure 4

**Western Blot**	Oxt			Inhibitor			Inhibitor+Oxt			
	mean	SEM	p value[a]	mean	SEM	p value[a]	mean	SEM	p value[a]	p (over inhibitor)[a]
Figure 4A - YM	1.402	0.0954	0.0072	0.5655	0.0450	<0.0001	0.6457	0.0857	0.0017	0.4272
Figure 4B - U0126	1.328	0.0720	0.0027	0.916	0.1432	0.5317	1.379	0.0461	0.0005	0.0088
Figure 4C - GF 2µM	1.199	0.0653	0.0376	1.058	0.0503	0.4513	0.908	0.1116	0.768	0.4636
Figure 4C - GF100nM							0.973	0.0762	0.4005	0.2888

[a] Student *t*-test

Table S5. Mean values, SEM, number of samples and p values of data displayed in Figure 5

	Oxtr$^{+/+}$ *(mean±SEM, N)*	*Oxtr*$^{-/-}$ *(mean±SEM,* N)	p value Student *t*-test
**Electrophysiology – Resting membrane potential**			
Figure 5A	-49.78±1.556 N=8	-51.33±1.641 N=9	0.4975
**Electrophysiology – Chemical LTP**			
Figure 5B - frequency			
pre-gly	1±0,09 N=16	1±0.07 N=15	1-way ANOVA<br>Dunn's Method*:*<br>*Oxtr*$^{+/+}$=0.004<br>*Oxtr*$^{-/-}$=0.025
15 post	1,26±0,20 N=8	0.97±0.18 N=8	
30 post	2,57±0.47(**) N=10	2.03±0.52 (*) N=7	
45 post	1.55±0.26 N=8	1.52±0.23 N=9	
Figure 5C – amplitude			
pre-gly	1±0.071 N=16	0.95±0.06 N=15	1-way ANOVA<br>Holm-Sidak method:<br>*Oxtr*$^{+/+}$=0.026<br>*Oxtr*$^{-/-}$=0.033
15 post	1.037±0.09 N=8	1.07±0.13 N=8	
30 post	1.46±0.26(*) N=10	1.34±0.19(*) N=7	
45 post	1.18±0.11 N=8	0.94±0.07 N=9	
**Electrophysiology – mEPSCs**			
Figure 5E (frequency)	1.871±0.32 N=9	2.776±0.24 N=15	0.037
Figure 5F (mean amplitude) (cumulative distribution)	22.48±2.24 N=9	25.08±1.62 N=15	0.349<br>*KS* test: p=0.22
Figure 5G (quantal charge)	68.72±12.02 N=9	88.45±9.71 N=15	0.107
**Electrophysiology - mIPSCs**			
Figure 5H (frequency)	1.42±0.24 N=9	1.19±0.13 N=15	0.202
Figure 5I (mean amplitude) (cumulative distribution)	21±1.99 N=9	18.69±2.10 N=14	0.013<br>*KS* test: p=0.004
Figure 5J (quantal charge)	204.11±39.80 N=9	93.05±17.68 N=14	0.022
**Electrophysiology – E/I ratio**			
Figure 5K	1.34±035 N=8	3.49±0.54 N=14	0.006

SUPPLEMENTAL EXPERIMENTAL PROCEDURES

Animals

Oxtr$^{+/+}$ and *Oxtr*$^{-/-}$ mice, rederived on a C57BL/6 genetic background (Charles Rivers, Calco, Italy), were stabulated in standard conditions, with *ad-libitum* access to food and water. Colony propagation has been carried out by heterozygous mating and litters were genotyped by PCR. Every animal procedure used was in strict accordance with standard ethical guidelines (European Community Guidelines on the Care and Use of Laboratory Animals 2010/63/EU) and the Italian legislation on animal experimentation (D.Lvo 116/92).

Primary hippocampal cultures

Embryonic day 18 dissociated hippocampal neurons were obtained from *Oxtr*$^{+/+}$ and *Oxtr*$^{-/-}$ timed pregnant mice as described by Kaech and Banker (Kaech and Banker, 2006), with slight modifications. Briefly, tissue dissociation was carried out with an enzymatic treatment (0.25% trypsin for 20 min at 37°C) followed by mechanic dissociation with a fire-smoothed Pasteur pipette. Dissociated cells were plated (30 000 cells/cm^2) in poly-L-lysine coated multiwell dishes containing Neurobasal medium (Life Technologies) additioned with B27 supplement (2% v/v; Life Technologies), L-glutamine (2mM), penicillin/streptomycin (100U/ml) and 25µM Glutamate. Five hours after plating the medium was replaced with a glutamate-free one to avoid excitotoxicity. Neurons were then maintained at 37°C in humidified atmosphere (95% air and 5% CO_2), and half of the medium was refreshed once a week.

Electrophysiology

Patch electrodes (GB150F-8P with filament, Science Products) are pulled from hard borosilicate glass on a Brown-Flaming P-87 puller (Sutter Instruments, Novato, CA, USA) and fire polished to a tip diameter of 1-1.5 µM and an electrical resistance of 4-6 MΩ.

Excitatory and inhibitory currents have been analyzed in DIV14 hippocampal neurons by whole-cell patch-clamp recordings of EPSCs and IPSCs in miniature using a Multiclamp 700A amplifier (Molecular Devices) and pClamp-10 software (Axon Instruments, Foster City, CA). Recordings were carried out in voltage-clamp mode in the presence of tetrodotoxin (TTX, 1 µM). Currents were sampled at 5 kHz, filtered at 2-5 kHz and analyzed (off-line) with Clampfit-pClamp 10.2 software. For the evaluation of the E/I balance, mEPSCs or mIPSCs were recorded using an internal solution containing CsGluconate (130mM), CsCl (8mM), NaCl (2mM), HEPES (10mM), EGTA (4mM), MgATP (4mM) and Tris-GTP (0.3mM), pH 7.4. The external solution (Krebs-Ringer's-HEPES - KRH) contained NaCl (125mM), KCl (5mM), $MgSO_4$ (1.2mM), KH_2PO_4 (1.2mM), $CaCl_2$ (2mM), glucose (6mM) and HEPES-NaOH (25mM), pH 7.4. The E/I ratio was calculated by dividing mEPSCs and mIPSCs frequencies measured in the same neuron. Thresholds were set at 8pA for mEPSCs and at 6pA for mIPSCs.

For the chemical LTP experiment (Fossati et al., 2015; Menna et al., 2013), basal excitatory events were monitored using an intracellular solution of KRH containing TTX (0.5µM), bicuculline (20µM, Tocris) and strychnine (1µM, Sigma-Aldrich). Glycine (100µM, Sigma-Aldrich) was subsequently applied for 3 min at room temperature in Mg^{2+}-free KRH containing TTX, bicuculline and strychnine and potentiation of EPSCs currents was recorded from 15 to 60 min after glycine delivery.

For cell-attached experiments the patch pipette was filled with the bath solution (in mM: 140 NaCl, 0.5 $MgCl_2$, 10 HEPES, 0.1 $CaCl_2$, 1 EGTA, pH 7.4) with the addition of 5 mM TEA and 100 □M DIDS to minimized K^+ and CLC ionic channel activity. Patch pipette tip was dipped in the described solution and back filled with the same solution supplemented with 10 □M GABA immediately before the experiment. Patches showing channel activity in the first 1-2 min after reaching the tight seal cell attached mode were discharged. Channel activity was observed using a step protocol from -80 to +80 mV pipette voltage in 20 mV steps and 400 ms duration. Routinely after 15 min of current recordings the membrane patch was broken in current-clamp mode to monitor the cell resting potential. Although the electrode contained an inappropriate solution for whole cell experiments, the measured resting potential, obtained at the end of each cell attached experiment, matched the values obtained using gramicidin perforated patch or with an electrode filled with the intracellular-like solution for whole cell recording (K-aspartate, 120mM; NaCl, 10mM; EGTA, 10mM; $MgCl_2$, 2mM; $CaCl_2$, 4mM; MgATP, 3mM; Na_2 GTP, 0.2mM; HEPES–KOH, 10mM, pH 7.2.). The amplitude of GABA-induced $GABA_A$ receptor channel was measured at different test potentials and plotted on a current/voltage relationship after the voltage was adjusted according to the measured membrane potential. Linear fit of the experimental points indicate the current reversal potential and in the case of $GABA_A$ receptor, the chloride reversal potential.

Treatments

Oxytocin (Oxt) was purchased from Bachem, GABA and PMA from Sigma-Aldrich. The selective Gq inhibitor YM-254890 (Takasaki et al., 2004) was a generous gift of Jun Takasaki (Astellas Pharma Inc., Tsukuba, Japan), whereas the MEK inhibitor U0126 was purchased from Cell Signalling and the PKC inhibitor GF109203X from TOCRIS. All drugs were pre-diluted in Neurobasal or KRH media and then applied to neurons at the final concentration and at the indicated time.

Calcium imaging

Hippocampal neurons were loaded with the membrane-permeable fluorescent Ca^{2+} indicator Fura-2/AM (1 μM; Sigma-Aldrich) for 30 min at 37°C, 5% CO_2. Following dye-loading, the cells were thoroughly washed with KRH buffer, subsequently used as extracellular recording solution. From DIV6 on, 1 μM TTX was added to this extracellular recording solution. Neurons were placed into the recording chamber of an inverted microscope (Axiovert 100, Zeiss) and imaged through a 40x objective (Zeiss).

Fura-2/AM was excited at 380 nm and at 340 nm through a Polychrom V, (TILL Photonics GmbH) controlled by the TillVisION software 4.01. Emitted light was acquired at 505nm at 1Hz, and images collected with a CCD Imago-QE camera (TILL Photonics GmbH).

The fluorescence ratio *F*340/380 was used to express Ca^{2+} concentrations. This parameter was recorded in regions of interest (ROIs) corresponding to neuronal cell bodies, and analyzed along sequential images to follow temporal changes.

After a period of basal recordings, GABA was administered at 100μM. Increases in *F*340/380 ratio (Δ*F*340/380) higher than 0.05 units within 15s after drug administration were considered reliable Ca^{2+} responses. After GABA recordings, neurons were washed with KRH and let to recover for a few minutes, before administering KCl (50mM) to identify viable neurons. Neurons responding to depolarization delivery with a Δ*F*340/380 smaller than 0.08 units were excluded from the analysis.

For the study of Oxt's action, a first GABA administration was followed by a short (2 min) recovery time. Subsequently, 100nM Oxt (or vehicle) was administered and left in the bath for 10 min, then a second GABA challenge was delivered to neurons. The percentage change in the Δ*F*340/380 of the second response with respect to the first one was scored for each individual neuron recorded.

GFP-transfection, imaging and morphological analysis

Oxtr$^{+/+}$ and *Oxtr*$^{-/-}$ neurons were seeded on glass coverslips at 25,000 cells/cm^2, and transfected at DIV7 with a GFP-expressing vector using Lipofectamine2000 (Life Technologies). For each well of a 12-well dish, 2.5 μg of DNA were used. Neurons were maintained until DIV17, then fixed with 4% paraformaldheyde-4% sucrose (w/v) and imaged with a 63x objective using a 510LSM Meta laser scanning confocal microscope (Zeiss). Focal planes were stacked together in a projection, then dendritic spines were counted manually. For each genotype 5-6 neurons were analyzed. Dendritic length and spine morphology parameters: Spine length (L), head diameter (H), and neck width (N) were measured using the ImageJ software (imagej.nhi.gov/ij/), then spines were assigned to different subclasses according to NeuronStudio software criteria: Mushroom spines: H/N>1.1μm and H>0.35 μm; Thin spines: H/N>1.1μm and H<0.35 μm or H/N<1.1μm and L/H>2.5 μm; Stubby spines: H/N<1.1μm and L/H<2.5 μm.

RNA extraction, cDNA synthesis, and Real-Time PCR

Neurons' RNA content was isolated using the Nucleospin RNA kit (Macherey-Nagel), following manufacturer's instructions. For each sample 500 μg of total RNA were retrotranscribed using the SuperScript III Reverse Transcriptase Kit (Life Technologies) according to manufacturer's instructions. For quantitative Real-Time PCR cDNA samples were amplified in triplicate, using 20 ng per replicate, with appropriate Taqman® Gene Expression Assay' s probes (KCC2: Slc12a5 #Mm00803929_m1; NKCC1: Slc12a2 #Mm00436554_m1; Oxtr: #Mm01182684_m1; Life Technologies). The housekeeping hypoxantine phosphoribosyltransferase 1 (HPRT-1) gene was used as reference gene and amplified in parallel using its specific TaqMan® Assay (Hprt1 #Mm00446968_m1; Life Technologies). Real-Time PCR was performed using the ABI Prims™ 7000 Sequence Detection System (Applied Biosystems). Results were elaborated with the ABI Prism1.2.3 software (Applied Biosystems) using the $2^{-\Delta\Delta Ct}$ method. Target genes (Kcc2, Nkcc1 and Oxtr) were normalized on the reference gene (Hprt-1) and compared with the calibrator (DIV1, set to 1) for each experiment.

Surface biotinylation assay

Neurons at DIV4 were transferred to KRH buffer for 10 min, then Oxt (final concentration 100nM), or KRH were incubated for subsequent 10 min. Treatments were stopped with ice-cold KRH, after which neurons were incubated for 15 min at 6°C with KRH containing EZ-Link-Sulfo-NHS-LC-biotin (1mg/ml). Biotin excess was quenched with two 10-min washes with a 50mM Glycine-KRH buffer solution (adjusted to maintain physiological osmolarity). Neurons were lysed in ice-cold RIPA buffer (NaCl 150mM, Tris-HCl 50mM, pH 7.4, EDTA 1mM, Triton X-100 1% and NP-40 1%) containing proteases and phosphatases inhibitors (Roche), and protein content was assessed by a DC protein assay (Bio-Rad). Equal amounts of proteins were loaded on Streptavidin beads (Ultralink Streptavidin Resin, Pierce) and left to rotate on a wheel for 16 hours. After a brief centrifugation, supernatants containing the non-biotinylated proteins were removed, beads were washed three times with PBS and finally biotinylated proteins were collected by adding 3x-Laemli Buffer (Tris-HCl 375mM, pH 6.8, glycerol 20% w/v, SDS 9% w/v, β-mercaptoethanol 10% v/v and Bromophenol Blue 0.05% w/v) to the beads and heating at 95°C for 3 min. Proteins were then separated by SDS-PAGE and processed for western blotting as described below.

Western blotting

Neurons were lysed in ice-cold RIPA buffer with a Protease Inhibitor Cocktail (Sigma) and Phosphatase Inhibitors (Roche). Protein concentration was determined with the DC protein assay kit (Bio-Rad) and samples were diluted in 3x-Laemli buffer. For Western blotting, 2-4 neuronal preparations were used and in each experiment neuronal samples derived from three independent wells were run for each data point.

Protein samples were separated by SDS-PAGE and then transferred onto a nitrocellullose membrane (GE Healthcare). Unspecific binding sites were saturated by incubation with BSA (5% w/v) for 1 hour at 37°C. Proteins of interest were probed with the proper primary antibody and revealed by secondary antibodies conjugated with infrared-emitting-fluorophores. Signals were detected and quantified using an Odissey scanner (Li-Cor) controlled by the ImageStudio software (Li-Cor). All the antibodies used and the respective suppliers and working dilutions are listed in the table below.

Protein MW (kDa)	Primary Antibody	Secondary Antibody
140	Rabbit Anti-Kcc2 (gift from *C. Rivera*) 1:3000	IRDye® 800CW Goat Anti-Rabbit (*Li-Cor*) 1:2500
140	Rabbit Anti-p-Ser940Kcc2 (*Rockland*) 1:1000	
42-44	Rabbit Anti-ERK1/2 (*Cell Signalling*) 1:1000	
42-44	Mouse Anti-p-ERK1/2 (*Cell Signalling*) 1:1000	IRDye® 680RD Goat Anti-Mouse (*Li-Cor*) 1:5000
55	Mouse Anti-β3-tubulin (*Promega*) 1:1000	
95	Mouse Anti-Transferrin Receptor (*Invitrogen*) 1:1000	

Primary and Secondary antibodies used for western blotting analysis. The molecular weight (MW) of expected bands is reported in the first column. Antibody suppliers are indicated in brackets. Working dilutions of each antibody is also reported.

Statistical analysis

For electrophysiology and Ca^{2+} imaging experiments, data were obtained from at least three different neuronal preparations. For qRT-PCR and Western blotting, replicates consisted in different wells coming from 2-4 independent neuronal preparations. Statistical analysis was performed with GraphPad Prism 5.0 (GraphPad Software, Inc.).

For experiments where *Oxtr*$^{+/+}$ and *Oxtr*$^{-/-}$ were compared repeatedly over time, 2-way ANOVA was used, with "time" and "genotype" as variables, followed by Bonferroni’s *post hoc* test. For direct comparisons of two data sets, Student’s *t*-test was used, whereas One-sample *t*-test was performed to assess significant differences of datasets from a fixed

control value (i.e. 1.00 or 100%). LTP experiments were analyzed by parametric or non parametric 1-way ANOVA followed by Holm-Sidak or Dunn's *post hoc* tests, respectively. To compare cumulative distributions Kolmogorov-Smirnov (*KS*) test was used. For all the experiments, results were deemed statistically significant when $p<0.05$.

SUPPLEMENTAL REFERENCES

Fossati, G., Morini, R., Corradini, I., Antonucci, F., Trepte, P., Edry, E., Sharma, V., Papale, A., Pozzi, D., Defilippi, P., *et al.* (2015). Reduced SNAP-25 increases PSD-95 mobility and impairs spine morphogenesis. Cell Death Differ *22*, 1425-1436.

Kaech, S., and Banker, G. (2006). Culturing hippocampal neurons. Nature protocols *1*, 2406-2415.

Menna, E., Zambetti, S., Morini, R., Donzelli, A., Disanza, A., Calvigioni, D., Braida, D., Nicolini, C., Orlando, M., Fossati, G., *et al.* (2013). Eps8 controls dendritic spine density and synaptic plasticity through its actin-capping activity. EMBO J *32*, 1730-1744.

Takasaki, J., Saito, T., Taniguchi, M., Kawasaki, T., Moritani, Y., Hayashi, K., and Kobori, M. (2004). A novel Galphaq/11-selective inhibitor. J Biol Chem *279*, 47438-47445.
